# *Acinetobacter stercoris* sp. nov. isolated from output source of a mesophilic german biogas plant with anaerobic operating conditions

**DOI:** 10.1007/s10482-021-01517-7

**Published:** 2021-02-16

**Authors:** Dipen Pulami, Thorsten Schauss, Tobias Eisenberg, Jochen Blom, Oliver Schwengers, Jennifer K. Bender, Gottfried Wilharm, Peter Kämpfer, Stefanie P. Glaeser

**Affiliations:** 1 Institut für Angewandte Mikrobiologie, Justus-Liebig-Universität Giessen, 35392 Giessen, Germany; 2 Department of Veterinary Medicine, Hessian State Laboratory, Giessen, Germany; 3 Institute for Bioinformatics and Systems Biology, Giessen, 35392 Giessen, Germany; 4 Division of Nosocomial Pathogens and Antibiotic Resistances, Wernigerode Branch, Robert Koch Institute, 38855 Wernigerode, Germany; 5 Project group P2, Wernigerode Branch, Robert Koch Institute, 38855 Wernigerode, Germany

**Keywords:** *Acinetobacter stercoris*, 16S rRNA, Genome, Biogas plant digestate

## Abstract

The Gram-stain-negative, oxidase negative, catalase positive strain KPC-SM-21^T^, isolated from a digestate of a storage tank of a mesophilic German biogas plant, was investigated by a polyphasic taxonomic approach. Phylogenetic identification based on the nearly full-length 16S rRNA gene revealed highest gene sequence similarity to *Acinetobacter baumannii* ATCC 19606^T^ (97.0%). Phylogenetic trees calculated based on partial *rpoB* and *gyrB* gene sequences showed a distinct clustering of strain KPC-SM-21^T^ with *Acinetobacter gerneri* DSM 14967^T^ = CIP 107464^T^ and not with *A. baumannii*, which was also supported in the five housekeeping genes multilocus sequence analysis based phylogeny. Average nucleotide identity values between whole genome sequences of strain KPC-SM-21^T^ and next related type strains supported the novel species status. The DNA G + C content of strain KPC-SM-21^T^ was 37.7 mol%. Whole-cell MALDI-TOF MS analysis supported the distinctness of the strain to type strains of next related *Acinetobacter* species. Predominant fatty acids were C_18:1_ ω9c (44.2%), C_16:0_ (21.7%) and a summed feature comprising C_16:1_ ω7c and/or iso-C_15:0_ 2-OH (15.3%). Based on the obtained genotypic, phenotypic and chemotaxonomic data we concluded that strain KPC-SM-21^T^ represents a novel species of the genus *Acinetobacter*, for which the name *Acinetobacter stercoris* sp. nov. is proposed. The type strain is KPC-SM-21^T^ (= DSM 102168^T^ = LMG 29413^T^).

**Supplementary Information:**

The online version of this article (10.1007/s10482-021-01517-7) contains supplementary material, which is available to authorized users.

## Introduction

The genus *Acinetobacter* is highly diverse (Touchon et al. 2014) and was first described by Brisou and Prévot (1954). Members of this genus are Gram-negative coccobacilli, non-motile, non-spore forming, aerobic, oxidase negative and catalase positive bacteria. This genus comprises non-fermentative bacteria, which can survive under different environmental conditions for extended periods through a wide temperature range. Over the past decades, some species of this genus have emerged as significant nosocomial and opportunistic pathogens causing outbreaks of colonization and infection, especially in critically ill patients with impaired immunity (Dijkshoorn et al. 2007; Peleg et al. 2008; Towner 2006; Visca et al. 2011). Accumulation of antibiotic resistances in *Acinetobacter* spp. is an increasing problem for the global public health (Visca et al. 2011). *Acinetobacter baumannii* represents one of the “ESKAPE pathogens” which can cause life-threatening nosocomial infections and can harbor several drug resistance mechanisms (Rice 2008; Bush and Jacoby 2010). At the time of writing, the genus *Acinetobacter* comprised 59 distinct species with validly published names (https://lpsn.dsmz.de/genus/acinetobacter; Parte 2018), as well as several species and genomic species without validly published names. Most of the species of *Acinetobacter* were obtained exclusively from human clinical specimens (Nemec et al. 2001, 2003, 2010, 2011, 2015, 2016, 2017). However, others were isolated from environmental sources, such as activated sludge (Carr et al. 2003), wetlands (Anandham et al. 2010), forest soil (Kim et al. 2008), seawater (Di Cello et al. 1997; Vaneechoutte et al. 2009), dumpsites (Malhotra et al. 2012), wastewater (Vaz-Moreira et al. 2011), freshwater (Li et al. 2014; Radolfova-Krizova et al. 2016), cotton and soil (Nishimura et al. 1988; Choi et al. 2013). Furthermore, Rafei et al. (2015) reported as many as 30 putative novel species of *Acinetobacter* in a non-human epidemiological study in Lebanon, which suggested that this genus is geographically more distributed than originally supposed.

In an attempt to isolate carbapenem-resistant bacteria released from biogas plants (anaerobic processing condition) digestates into the environment, strain KPC-SM-21^T^ was isolated in October 2013 from the digestate collected from one of the studied German biogas plants (Schauss et al. 2015). Here, detailed phenotypic, genotypic and chemotaxonomic studies of strain KPC-SM-21^T^ were performed and the taxonomic status was concluded. Based on morphological, physiological, biochemical and genotypic characteristics obtained on the notion of a polyphasic approach, we propose a novel species of the genus *Acinetobacter* with strain KPC-SM-21^T^ as type strain. Besides, genes encoding antibiotic resistance, virulence and bacteriophages were identified, and survival of this strain in anaerobic condition was also investigated.

## Materials and methods

### Isolation and culture condition

The studied strain was isolated in 2013 from a digestate sample obtained from the final storage tank of a biogas plant (BGP-1) located in the North of Hesse, Germany. The input material of the biogas plant was composed of 54% slurry (20:1 cattle to pig) and 46% manure (6:1 cattle to chicken) and corn and forage rye as co-substrates (Schauss et al. 2015). The biogas plant contained a continuous stirred tank reactor (CSTRs) typical for German on farm small scale systems with a two stage mesophilic digestion process (T = 44 °C). Strain KPC-SM-21^T^ was cultured by a selective pre-enrichment method which was applied to culture carbapenem-resistant bacteria from the collected output material. Briefly, 10 g digestate of the storage tank was incubated directly in 90 mL sterile lysogen broth (LB, Sigma-Aldrich) containing 1 mg L^−1^ meropenem (MER: C_17_H_25_N_3_O_5_S3H_2_O, Sigma-Aldrich). After 24 h of incubation at 37 °C under continuous shaking at 180 rpm, 10 µL of the pre-enrichment culture was streaked on CHROMagar KPC (CHROMagar, France). The agar plate was incubated for 24 h at 37 °C. Among morphologically different colonies grown on the agar plates, one of separately lying cream-colored colony represented strain KPC-SM-21^T^ which was obtained as pure culture after multiple transfer steps of single colony following singular streaking on CHROMagar KPC. After purification, fresh biomass of strain KPC-SM-21^T^ was cultured on LB agar containing 1 mg L^−1^ meropenem and suspended in sterile Gibco newborn calf serum (NBCS, ThermoFisher Scientific) and stored at − 20 °C and − 80 °C for long-term preservation.

### Phylogenetic identification

Bacterial cell lysate and 16S rRNA gene sequencing for molecular analyses was generated and performed as described by Schauss et al. (2015). Universal 16S rRNA gene targeting primers [8F: 5′-AGAGTTTGATCCTGGCTCAG-3′ and 1492R: 5′-CGGTTACCTTGTTACGACTT-3′; (Turner et al. 1999)] were used for PCR and primers 27F [5′-GAGTTTGATCMTGGCTCAG-3′; (Lane 1991)] and E786F [5′-GATTAGATACCCTGGTAG-3′; (Baker et al. 2003)] for Sanger sequencing performed at LGC Genomics (Berlin, Germany). The partial gene sequences were corrected in MEGA7 (Kumar et al. 2016) based on electropherograms and concatenated to a nearly full-length 16S rRNA gene sequence. Next related type strains were determined using the EzBioCloud 16S rRNA gene identification system (Yoon et al. 2017). The phylogenetic relationship of KPC-SM-21^T^ to the type strains of the genus *Acinetobacter*, including several genomic species and multiple species without validly published names, was studied based on nearly complete 16S rRNA gene sequences. 16S rRNA genes sequences of all representatives of this genus were retrieved from the NCBI database (https://www.ncbi.nlm.nih.gov/nucleotide/) and aligned with ClustalW (Thompson et al. 1994) provided in MEGA7. The phylogenetic tree was constructed using the maximum-likelihood method (ML; Felsenstein 1981) based on the Kimura 2-parameter model (Kimura 1980). The consistency of the phylogenetic tree was investigated by 100 resamplings (bootstrap analysis; Felsenstein 1985). Moreover, phylogenetic analyses with higher resolution were performed based on protein coding sequences including the RNA polymerase *β*-subunit (*rpoB*) and DNA gyrase subunit B (*gyrB*) genes as described previously (Nemec et al. 2009; Krizova et al. 2014). Alignments of the nucleotide sequences of each gene were performed based on the respective correct open reading frame (ORF). Pairwise nucleotide sequence similarities were determined with the p-distance method implemented in MEGA7. Phylogenetic analyses were performed using the ML method based on the General Time Reversible (GTR; Nei and Kumar 2000) model for nucleotide and the Jones–Thornton-¯Taylor matrix-based (JTT; Jones et al. 1992) model for amino acid sequence based analysis. Multilocus sequence analysis (MLSA) was performed based on genes used in the multilocus sequence typing (MLST) scheme (Pasteur; https://pubmlst.org/abaumannii/) for *A. baumannii* (Diancourt et al. 2010). Partial sequences of six housekeeping genes were used for MLSA analysis. The genes code for CTP synthase (PyrG), 60-KDa chaperonin (Cpn60), citrate synthase (GltA), homologous recombination factor (RecA), 50S ribosomal protein L2 (RplB) and the beta-subunit of the RNA polymerase (RpoB), respectively. The *rpoB* gene fragment used in the MLSA approach [spanning nucleotide positions 1681–2136 of the *rpoB* gene of *A. baumannii* CIP 70.34^T^ (DQ207471)] was different from that applied in the *rpoB* gene based phylogeny. Full-length sequences of these housekeeping genes for type strains of *Acinetobacter* species were obtained from the NCBI database. Sequences were aligned based on the correct ORF and concatenated in the following order: *pyrG* (297 nt), *cpn60* (405 nt), *gltA* (483 nt), *recA* (361 nt), *rplB* (330 nt) and *rpoB* (456 nt) based on their respective sizes. The gene encoding for elongation factor G (FusA), mentioned in the MLST scheme, was not used in MLSA, since the amplification result of the *fusA* gene was unsatisfactory. The evolutionary history was inferred using the ML method based on the GTR model (Nei and Kumar 2000).

### Genome sequencing, core genome based phylogeny and genome‐wide analysis

A draft genome sequence of strain KPC-SM-21^T^ was generated by Illumina short read sequencing (read out 2 × 300 bp, MiSeq benchtop sequencer) followed by sequence reconstruction using the A5-miseq assembly pipeline. Genome sequence based analyses were performed in EDGAR 2.3 (Blom et al. 2016). The genome sequence of strain KPC-SM-21^T^ and genome sequences of *Acinetobacter* species (validly published) type strains and strains representing distinct genomic species with provisional designation or *Acinetobacter* species without names standing in nomenclature were obtained from the NCBI database and integrated into an EDGAR project. The BLAST search of the 16S rRNA gene sequence of strain KPC-SM-21^T^ showed 99.7% similarity to the 16S rRNA gene of *Acinetobacter* sp. Marseille-Q1620 (LR782267.1). Therefore, the genome of *Acinetobacter* sp. Marseille-Q1620 (NZ_LR782267) was also included to determine the taxonomic position of strain KPC-SM-21^T^.

The taxonomic status at the whole genome level was assessed by calculating average nucleotide identity (ANI) values. An ANI matrix was calculated in EDGAR based on the BLASTN comparison of the genome sequences as described by Goris et al. (2007). A core genome based phylogenetic analysis was calculated in EDGAR following a stepwise alignments of each core gene set using MUSCLE (implemented in EDGAR 2.3) the final alignments were concatenated to one huge alignment, which included shared genes of the genome of strain KPC-SM-21^T^, the *Acinetobacter* reference genomes and the genome of *Moraxella lacunata* NBRC 102154^T^ (NZ_BCUK00000000) which was used as outgroup. Thereafter, a core genome based phylogenetic analysis was computed using the FastTree software (http://www.microbesonline.org/fasttree/) to generate approximately-maximum-likelihood phylogenetic trees (Price et al. 2009, 2010) implemented in EDGAR 2.3. The genome-based circular plot was generated with BioCircos (Cui et al. 2016) implemented in EDGAR 2.3. Furthermore, EDGAR 2.3 and VFDB (virulence factor database; http://www.mgc.ac.cn/VFs/) were used to identify resistance and virulence associated genes. Genomic islands (GIs) were searched with IslandViewer4 (Bertelli et al. 2017; Bertelli and Brinkman 2018). Potential phage-related genes of strain KPC-SM-21^T^ were identified using PHASTER (https://phaster.ca/; Zhou et al. 2011; Arndt et al. 2016).

### Matrix‐assisted laser desorption ionization time‐of‐flight mass spectrometry (MALDI-TOF MS)

For MALDI-TOF MS the strain was grown on Columbia agar with 5% sheep blood (SBA; Oxoid) for 24 h. The experiment was performed as described by Eisenberg et al. (2017). Biomass was transferred to steel targets using the direct transfer protocol according to the manufacturer’s instruction (MALDI Biotyper; Bruker Daltonics, Bremen, Germany). Analysis was performed on a MALDI-TOF MS Biotyper version 3.3.1.0; commercial database (DB 8468; BrukerDaltonics). The MALDI Biotyper real-time classification (RTC) software calculated obtained log score based on similarities between the observed results and stored database sets. Log scores of > 2.3 and > 2.0 were considered as species and genus level identifications, respectively. The identification was repeated three times to verify the original findings.

### Fatty acid analysis

Biomass for fatty acid analysis was harvested after growth on trypticase soy agar (TS agar; Becton Dickinson GmbH) at 30 °C for 48 h (exponentially growing cells). The analysis was performed as described by Kämpfer and Kroppenstedt (1996) using the Sherlock version 2.11, TSBA40 Rev. 4.1 for identification.

### Phenotypic characterization

Cell morphology and motility was observed under a Zeiss light microscope at a magnification of × 1000, using cells grown for three days at 25 °C on TS agar. Gram-staining was performed by the modified Hucker method according to Gerhardt et al. (1994). Cytochrome-c oxidase activity was tested using Bactident oxidase test strips (Merck) and catalase enzyme activity by testing formation of gas bubbles after dropping 3% (v/v) hydrogen peroxide (H_2_O_2_) onto overnight grown biomass on TS agar. The test of growth on different agar media and temperature-dependent growth was performed by suspending fresh biomass in 0.9% (w/v) sodium chloride (NaCl); turbidity standardized by 0.5 McFarland. The cell suspension was serially diluted up to 10^−5^ and 5 µL of each dilution were spotted on following media: TS agar, R2A agar (R2A; Oxoid), nutrient agar (NA; Becton Dickinson), malt agar (Merck), glycine arginine agar (Gly/Arg; Oxoid), CASO agar (Carl Roth), K7 [0.1% (w/v) of yeast extract, peptone, and glucose, agar (15 g L^−1^), pH 6.8], M65 medium (according to DSMZ), DEV agar (DEV; Merck), Luria Bertani (LB; Sigma-Aldrich), MacConkey agar (Oxoid), PYE [0.3% (w/v) yeast extract and 0.3% (w/v) casein peptone, agar (15 g L^−1^), pH 7.2)], nutrient broth (NU; Oxoid), marine agar (MA; Becton Dickinson) and SBA, respectively. Thereafter, all plates were incubated at 28 °C and growth was analysed after 7 days. For temperature-dependent growth the serially dilutions were spotted on TS agar plates which were incubated at 4, 10, 15, 20, 25, 28, 30, 37, 45, 50, and 55 °C, respectively, as described by Pulami et al. (2020). The growth was monitored after 24 h, 48 h, 3 and 7 days of incubation. Hemolysis test was performed as previously described by Nemec et al. (2016). The physiological characterization was performed as described by Kämpfer et al. (1991). Furthermore, strain KPC-SM-21^T^ was tested with the API 20 NE kit (BioMérieux) following the instructions of the manufacturer.

### Anaerobic growth test

The survival of strain KPC-SM-21^T^ and *A. baumannii* ATCC 19606^T^ under anaerobic conditions was investigated by taking strains pre-grown (overnight aerobically at 25 °C) on NA plates, and exposing them to anaerobic conditions using the Anaerocult A system (Merck) at the same temperature for 7 days. Thereafter, a loop of biomass was re-inoculated onto fresh NA, and growth was checked after overnight aerobic incubation at 37 °C. The ability of the strain to grow under anaerobic conditions was checked by direct exposure of streaked plates to anaerobic conditions using the Anaerocult A system at 25 °C for 7 days.

## Results and discussion

### Molecular and genome characteristics

**Fig. 1:**
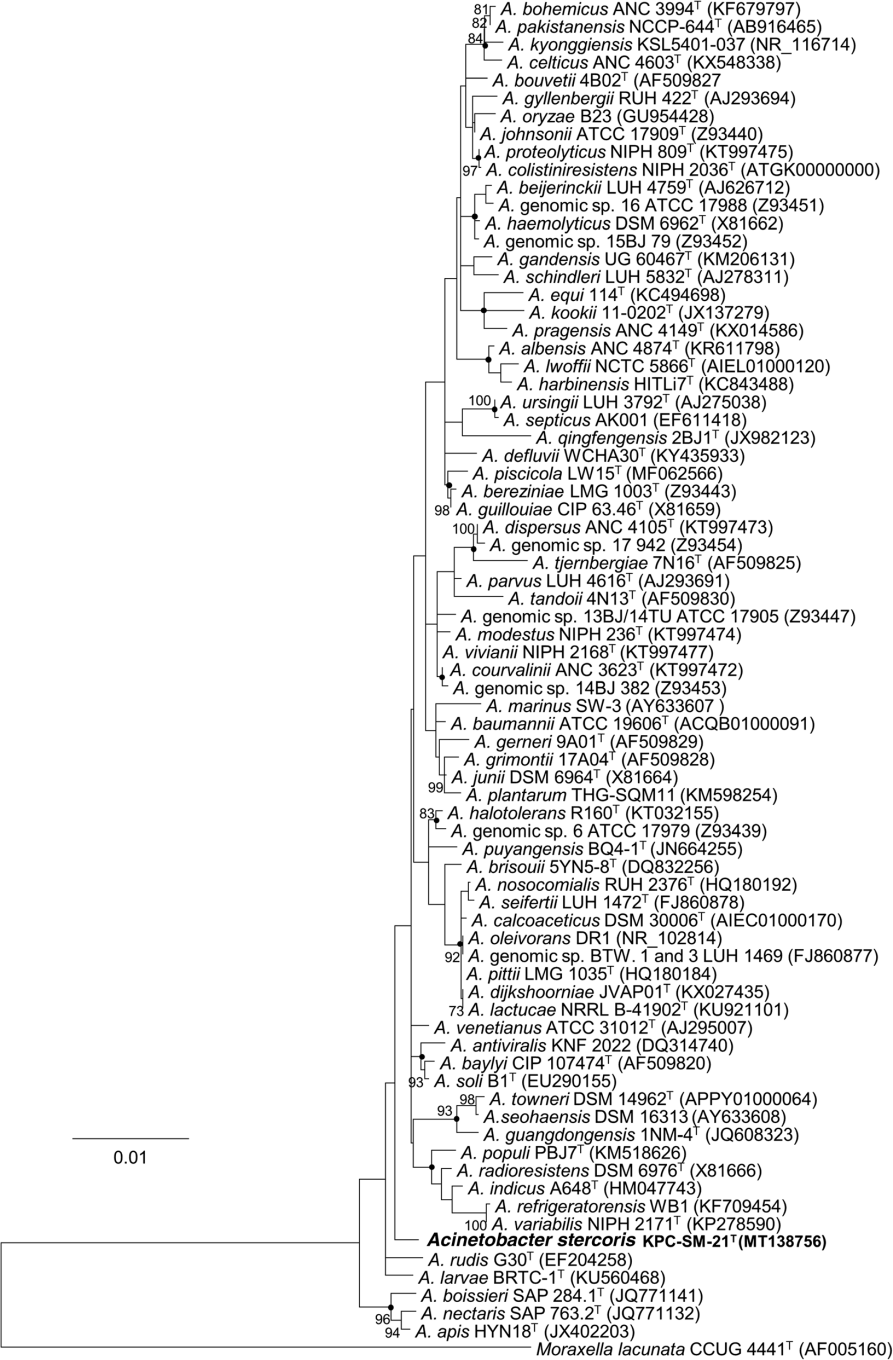
Phylogenetic placement of strain KPC-SM-21^T^ within the genus *Acinetobacter* based on nearly full-length 16S rRNA gene sequences. The maximum-likelihood tree was generated in MEGA7 and is based on nucleotide positions 28–1468 (according to *E. coli* numbering; Brosius et al. 1978). The respective gene sequence of the type strain of *Moraxella lacunata* was used as outgroup. Numbers at nodes represent bootstrap values (> 70%) based on 100 replications. Filled circles indicate nodes that were conserved in a tree generated with the neighbour-joining (NJ) method. GenBank accession numbers are given in parentheses. Bar, 0.01 substitutions per nucleotide position

**Fig. 2:**
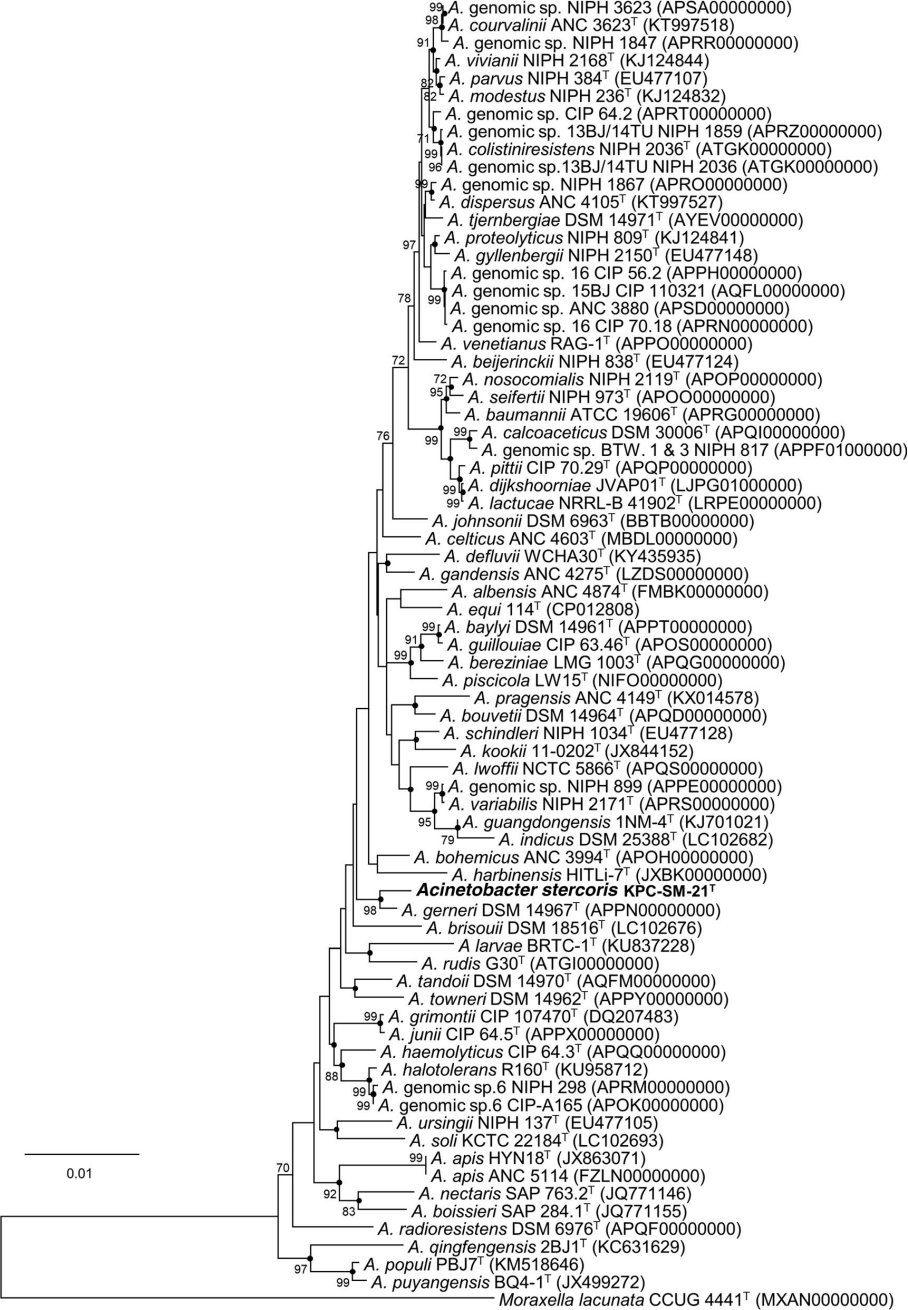
Phylogenetic placement of strain KPC-SM-21^T^ within the genus *Acinetobacter* based on nucleotide sequences of concatenated variable zones of the *rpoB* gene. The tree was calculated with the ML method based on 753 nucleotide positions in the final dataset. Numbers at nodes represent the percentage of replicate trees in which the associated taxa clustered together in bootstrap tests (100 replications). Only bootstrap values of 70% and above were shown. Filled circles indicate nodes that were also present in a tree generated with the NJ method. *Moraxella lacunata* NBRC 102154^T^ was used as outgroup. Bar, 0.01 substitutions per sequence position

The 16S rRNA gene sequence of strain KPC-SM-21^T^ obtained by Sanger sequencing was 1439 nucleotides in length, spanning gene termini 28 to 1468 [numbering according to the *Escherichia coli rrnB* (Brosius et al. 1978)], and initial phylogenetic assignment obtained by BLAST against the EzBioCloud database showed 97.0% similarity to *A. baumannii* ATCC 19606^T^. Sequence similarities to all type strains of *Acinetobacter* species were ≤ 97%. This indicated that strain KPC-SM-21^T^ represented a novel species, because all similarity values were below that of 98.65%, which was suggested by Kim et al. (2014) as a pre-requisite threshold to delineate a prokaryotic species. The ML tree based on 16S rRNA gene sequences was based on 1223 nucleotide positions. It showed the placement of strain KPC-SM-21^T^ in a separate branch within the genus *Acinetobacter* without a distinct clustering to any of the other investigated strains including all type strains of the genus (Fig. 1). The *rpoB* based phylogenetic analyses included gene fragments spanning gene positions 2917–3267 (zone1) and 3322–3723 (zone2), respectively. Gene termini were given according to the gene sequence obtained from *A. baumannii* CIP 70.34^T^ (DQ207471, La Scola et al. 2006). The nucleotide sequence of the concatenated variable zones of the *rpoB* gene of strain KPC-SM-21^T^ showed highest sequence similarity to *A. gerneri* DSM 14967^T^ (91.1%), followed by *A. guillouiae* CIP 63.46^T^ (86.9%) and *A. baylyi* DSM 14961^T^ (86.6%); the *rpoB* sequence similarity to *A. baumannii* ATCC 19606^T^ was lower (82.6%). The obtained *rpoB* nucleotide sequence similarity values were below 95% to tested next related type strains of the genus *Acinetobacter*. La Scola et al. (2006) and Narciso-da-Rocha et al. (2013) have suggested that *rpoB* gene sequence similarities below 95% represent distinct *Acinetobacter* species. The ML tree based on *rpoB* nucleotide (Fig. 2) and amino acid sequences (Fig. S1) showed that strain KPC-SM-21^T^ formed a distinct cluster with *A. gerneri* DSM 14967^T^ which was supported by high bootstrap values (> 70%). GyrB based phylogenetic analysis was performed with a gene region encompassing nucleotide positions 457–1209 (numbering according to *A. baumannii* ATCC 19606^T^ (Genome accession number: APRG00000000, Locus tag: 911_RS22805). The *gyrB* gene sequence based analysis also showed highest nucleotide sequence similarity with *A. gerneri* DSM 14967^T^ (85.2%). Sequence similarities with all other tested *Acinetobacter* sp. type strains were below 83.5%. The *gyrB* nucleotide sequence based phylogenetic tree also showed a distinct cluster of KPC-SM-21^T^ and *A. gerneri* DSM 14967^T^. However, this cluster was not supported with a high bootstrap value (Fig. S2). Similarly, the phylogeny based on amino acid sequences of *rpoB* (Fig. S1) and *gyrB* (Fig. S3) also showed the placement of strain KPC-SM-21^T^ in a separate branch within the genus *Acinetobacter.* The ML tree based on MLSA data placed strain KPC-SM-21^T^ in a separate branch beside other *Acinetobacter* sp. type strains (Fig. S4). Interspecies similarities of strain KPC-SM-21^T^ to other type strains was in the range of 82.7–89.4% (concatenated nucleotide sequences).

**Fig. 3:**
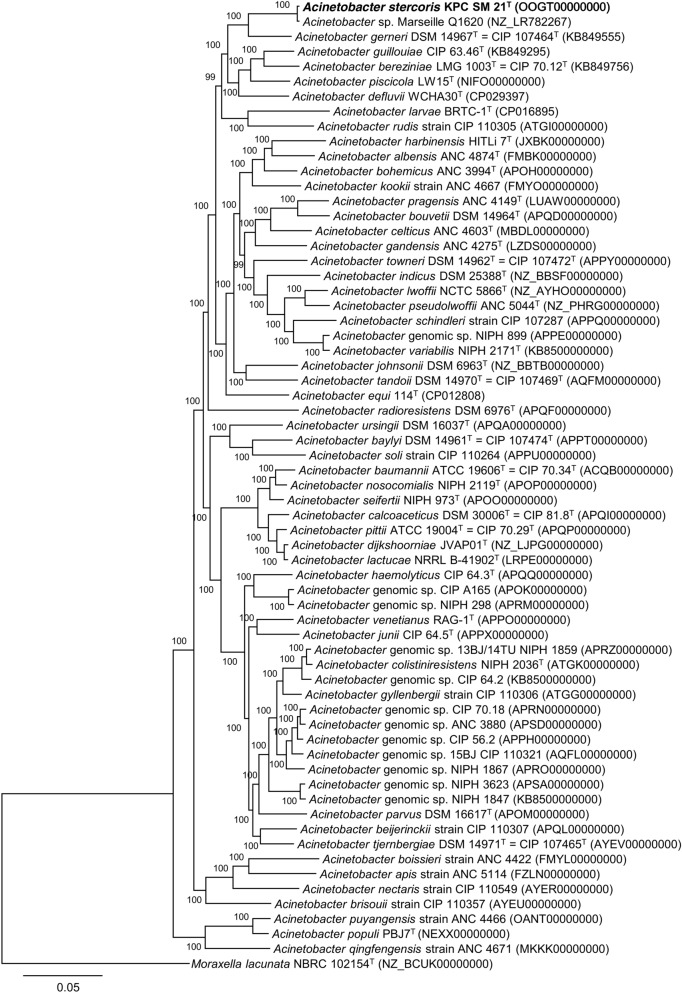
Phylogenetic tree based on 65 genomes, built out of a core of 668 genes per genome; 43,420 in total using EDGAR 2.3 (Blom et al. 2016), applying the FastTree software (http://www.microbesonline.org/fasttree/) to generate an approximately-ML phylogenetic tree (Price et al. 2009, 2010). The values at the branches show local support values in percentage computed by FastTree using the Shimodaira–Hasegawa test. The core has 720,855 amino acid residues per genome and 46,855,575 in total. The genome of *Moraxella lacunata* NBRC 102154^T^ (NZ_BCUK00000000) was used to root the tree. Bar, 0.05 substitutions per amino acid sequence residue

Prior to genome sequence-based analyses, the 16S rRNA gene sequence present in the genome sequence on contig OOGT01000238 (locus_tag: KPC_R004) was aligned with the Sanger sequenced 16S rRNA gene; both were identical. The draft genome sequence of strain KPC-SM-21^T^ (accession number OOGT01000000, Bioproject: PRJEB25537) had a total nucleotide length of 4.16 Mbp. The core genome-based phylogenetic tree (Fig. 3) showed distinct cluster of strain KPC-SM-21^T^ including *Acinetobacter* sp. Marseille-Q1620 with *A. gerneri *DSM 14967^T^, respectively. The relationship between strain KPC-SM-21^T^, *Acinetobacter* sp. Marseille-Q1620, *A. gerneri* DSM 14967^T^ and *A. baumannii* ATCC 19606^T^ at whole genome level was assessed by calculating average nucleotide identity (ANI) values in EDGAR 2.3. The ANI values were 98.3% (KPC-SM-21^T^ vs. *Acinetobacter* sp. Marseille-Q1620), 77.7% (KPC-SM-21^T^ vs. *A. gerneri* DSM 14967^T^) and 73.6% (KPC-SM-21^T^ vs. *A. baumannii* ATCC 19606^T^), respectively (Fig. S5). The core genome-based phylogeny and ANI values proved that strain KPC-SM-21^T^ and *Acinetobacter* sp. Marseille-Q1620 belonged to the same cluster of species and are genomically closely related. The ANI values against *A. gerneri* DSM 14967^T^ and *A. baumannii* ATCC 19606^T^ were below the threshold of ~ 95–96% proposed to discriminate between prokaryotic species (Richter and Rosselló-Móra 2009). The genomic DNA G + C content of strain KPC-SM-21^T^ was 37.7 mol%, which was similar to that of the two closely related type strains, 39.2 mol% for *A. baumannii* ATCC 19606^T^ and 37.9 mol% for *A. gerneri* DSM 14967^T^, respectively.

Therefore, on the basis of 16S rRNA gene, *rpoB* comparative analysis, *gyrB* phylogeny, and MLSA, strain KPC-SM-21^T^ was distinct from the type strains of *Acinetobacter* species with validly published names, genomic species with provisional designation or *Acinetobacter* species without names standing in nomenclature. Notably, ANI values and core genome-based phylogeny proved the high similarity between strain KPC-SM-21^T^ and *Acinetobacter* sp. Marseille-Q1620 below the threshold of prokaryotic species. The strains clustered with the type strain of *A. gerneri* which is represented by two genome sequences (*A. gerneri* DSM 14967^T^ and *A. gerneri* CIP 63.46^T^).

### Assignment by MALDI-TOF and fatty acid analysis

MALDI-TOF data confirmed the genotypic identification of strain KPC-SM-21^T^ as novel *Acinetobacter* species. The dendrogram based on MALDI-TOF data showed a distinct clustering of strain KPC-SM-21^T^ (Fig. S6) among type strains of next related *Acinetobacter* species. The average log score was 1.56, which was a non-reliable score that can be explained by absence of a close relative of KPC-SM-21^T^ in the database used. Therefore, and in a comparison with other species from the same genus, strain KPC-SM-21^T^ represented a distinct species of the genus *Acinetobacter* on the basis of MALDI-TOF data.

The predominant fatty acids of KPC-SM-21^T^ were C_18:1_ w9c (44.17%), C_16:0_ (21.67%) and summed feature 3* (15.34%) (containing C_16:1_ ω7c and/or iso-C_15:0_ 2-OH that was not determined by Sherlock version). The fatty acid pattern is typical for the genus *Acinetobacter* (Kämpfer et al. 1993; Kim et al. 2008; Vaz-Moreira et al. 2011). The presence of minor amounts of C_18:3_ ω6c (2.2%) differentiated strain KPC-SM-21^T^ from type strains of *A. baumannii*, *A. gerneri* and *A. guillouiae*, respectively. The details of the fatty acid profile is given in Table 1.

**Table 1:** Fatty acid composition of strain KPC-SM-21^T^ and selected *Acinetobacter* species

Fatty acids	1	2	3
C_12:0_	3.6	4.5	4.3
C_12:0_ 2-OH	4.9	2.0	4.7
C_12:0_ 3-OH	3.9	3.2	6
Summed feature 2*	4.2	3.1	1.3
Summed feature 3*	15.3	17.5	15.3
C_16:0_	21.7	27.8	19.6
C_17:1_ ω8c	(–)	3.1	(–)
C_18:3_ ω6c	2.2	(–)	(–)
C_18:1_ ω7c	(–)	(–)	1
C_18:1_ ω9c	44.2	38.9	41.9

The results of strain KPC-SM-21^T^ and *A. baumannii* ATCC 19606^T^ were from this study. Data for type strain of *A. gerneri* was adapted from Lee et al. (2009)

Strain: 1, KPC-SM-21^T^; 2, *A. baumannii* ATCC 19606^T^; 3, *A. gerneri* DSM 14967^T^ = KCTC 12415^T^. Values are percentage of total fatty acids. Values ≤ 1 are not shown. (−), Not detected

*Summed feature 2 in the MIDI system, contained iso-C_16:1_ I and/or C_14:0_ 3-OH

*Summed feature 3 in the MIDI system, contained C_16:1_ ω7c and/or iso-C_15:0_ 2-OH

### Phenotypic characteristics

Cells of strain KPC-SM-21^T^ were Gram-negative, oxidase negative, catalase positive and non-motile coccobacilli as typical for members of the genus *Acinetobacter*. The optimum growth temperature was 25–37 °C; growth occurred at 45 °C and 10 °C, but not at 50 °C and 4 °C. Growth at 45 °C differentiated strain KPC-SM-21^T^ from type strains of *A. gerneri* (Carr et al. 2003) and *A. guillouiae* (Nemec et al. 2010). Good growth occurred at 28 °C after 24 h on TS agar, R2A, NA, malt, Gly/Arg, CASO, K7, M65, DEV, LB, PYE, NU, and SBA. Very weak growth on MA, and no growth on MacConkey agar was observed. A zone of hemolysis was not formed on SBA. The outcome of microscopy, growth at different media and range of temperature are provided in supporting information (Fig. S7, S8 and S9). Strain KPC-SM-21^T^ grew on a broad range of carbon sources, and showed acidification of some sugars, as α-d-glucose, α-d-lactose, l-arabinose, d-xylose, d-cellobiose, α-d-melibiose and d-mannose. However, acid production from several sugars and sugar-related compounds was not observed. Physiological tests performed with 96 wells test panel (Kämpfer et al. 1991) resulted difference in comparison with the members of the genus *Acinetobacter*. Briefly, the ability to produce acid from α-d-melibiose, and assimilation of cis-aconitate, l-aspartate, l-histidine and l-tryptophan differentiated strain KPC-SM-21^T^ form *A. gerneri* 9A01^T^ = DSM 14967^T^. Formation of acid from d-glucose, d-mannose, α-d-melibiose, α-d-lactose, d-xylose and l-arabinose, and assimilation of cis-aconitate, l-phenylalanine and l-tryptophan differentiated the strain from members of *A. guillouiae* (genospecies 11). Lack of assimilation of trans-aconitate, l-arginine and l-leucine differentiated the strain from the members of *A. calcoaceticus-A. baumannii* (ACB) complex. The discriminating physiological characteristics are provided in Table 2.

**Table 2:** Physiological and metabolic characteristics of strain KPC-SM-21^T^ and selected species of the genus *Acinetobacter*

Characteristic	KPC-SM-21^T^	ACB complex	*A. guillouiae* (genospecies 11)	*A. gerneri*
Number of strains	1	73	7	1
*Acid production from*				
d-Glucose	+	89	0	100
d-Cellobiose	+	89	0	100
d-Mannose	+	88	0	100
α-d-Melibiose	+	89	0	0
α-d-Lactose	+	88	0	100
d-Xylose	+	89	0	100
l-Arabinose	+	89	0	100
*Assimilation of*				
Adipate	+	97	100	100
Azelate	w	97	100	100
cis-Aconitate	w	95	0	0
trans-Aconitate	−	93	0	0
4-Aminobutyrate	+	100	86	100
β-Alanine	+	93	100	100
l-Arginine	−	100	0	0
l-Aspartate	*+*	97	100	0
Citrate	+	100	57	100
Glutarate	+	97	100	100
l-Histidine	+	100	100	0
4-Hydroxybenzoate	+	95	86	100
l-Leucine	−	99	0	0
l-Phenylalanine	+	82	0	100
l-Tryptophan	+	93	0	0
Phenylacetate	+	85	71	100

All species with validly published names include the respective type strains. The results for KPC-SM-21^T^ were obtained in this study, while other data were adapted from Kämpfer et al. (1993) and Carr et al. (2003). +, positive; −, negative; w, weakly positive reaction

### Antibiotic resistance, virulence and phage associated genes

**Fig. 4:**
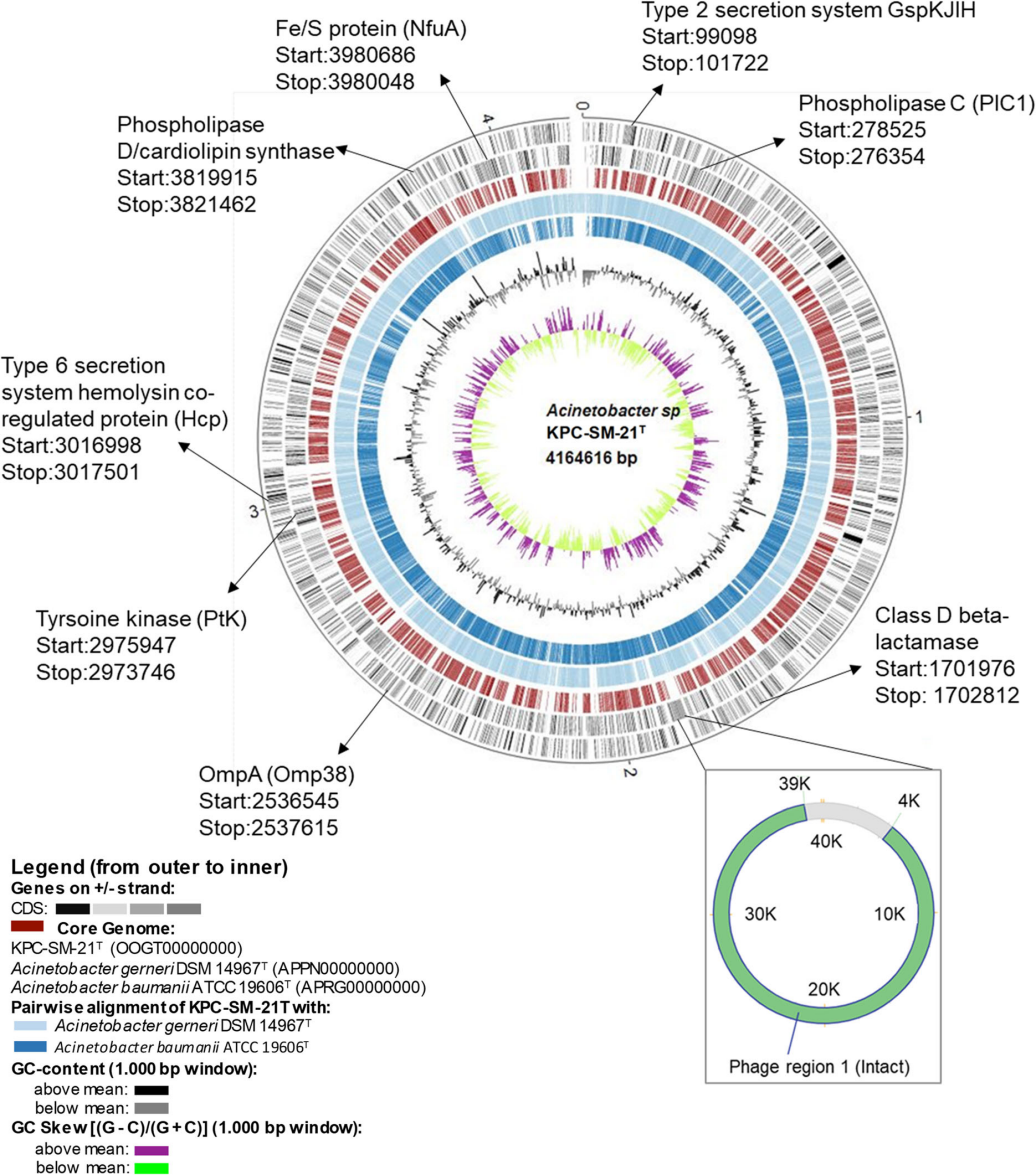
A circular plot obtained from the pairwise alignment of the genomes of strain KPC-SM-21^T^ (size given at the center of plot), *A. gerneri* DSM 14967^T^ and *A. baumannii* ATCC 19606^T^. The circular plot was generated with BioCircos (Cui et al. 2016) implemented in EDGAR 2.3 (Blom et al. 2016)

Although strain KPC-SM-21^T^ was isolated from non-clinical environment (output digestate of a biogas plant), it shared virulence related genes, for instance, those involved in immune evasion and cellular invasion, persistence, serum resistance, host cell lysis, inhibition of blood coagulation, in vivo survival and interspecies competition for host colonization previously reported among nosocomial *A. baumannii* strains (detailed in Table S2). The protein-protein BLAST (Blastp) of the metalloprotease (CpaA, Table S2) of strain KPC-SM-21^T^ shared 59% (99% query coverage) and 55.5% (99% query coverage) amino acid sequence homology with CpaA of *Acinetobacter* sp. TGL-Y2 (accession: WP_067658284) and *A. baumannii* (accession: WP_153566028). This gene was absent in *A. gerneri* DSM 14967^T^ (APPN00000000) which was a close relative of strain KPC-SM-21^T^, and also in the clinical strains ATCC 19606^T^ and ATCC 17978^T^ of *A. baumannii* isolated during middle of the last century (Tilley et al. 2014). Strain KPC-SM-21^T^ harboured an intrinsic *bla*_OXA−like_ Class D beta lactamase (Locus tag: KPC_0052) without transposition of insertion sequence element upstream this gene, and the strain also lacked potent acquired antibiotic resistance genes. As indicated by Perichon et al. (2014) the class D beta lactamase genes appeared to be intrinsic to several species of the genus *Acinetobacter*. Genomic islands (GIs) searched with IslandViewer4 showed absence of GIs with acquired resistance in the genome of strain KPC-SM-21^T^. Potential phage-related gene search in PHASTER showed five incomplete and fragmented phages integrated into the genome. Additionally, a phage with putative intact region (34.6 kb) available in contig NZ_OOGT01000008.1 of strain KPC-SM-21^T^ was found (Fig. 4 and Table S3). The intact phage region harboured segments that coded putative phage-like protein, putative head protein, putative tail protein, putative fiber protein and multiple hypothetical proteins, however lacked regions that code proteins responsible for termination, integration and lysis which are required for propagation inside the host bacterium (Casjens 2003; Canchaya et al. 2003; Labrie et al. 2010) (Fig. S10). This intact phage region shared 51.3% of proteins (data from PHASTER) with PHAGE_Acinet_YMC11/11/R3177 (GenBank accession: NC_041866) (Table S3).

### Survival in anaerobic conditions

Both strains, KPC-SM-21^T^ and *A. baumannii* ATCC 19606^T^, failed to grow under anaerobic conditions. However, both survived in anaerobic conditions on NA plates for a week at 25 °C, and thereafter grew well in aerobic conditions at 37 °C (data not shown). Even though the genus *Acinetobacter* is generally regarded as obligate aerobe, they can survive in different anaerobic or oxygen-limited environments, including anaerobic digesters (Supaphol et al. 2011; Baek et al. 2014; Jo et al. 2015). Recently Higgins et al. (2018) reported that *Acinetobacter* spp. survived the activated anaerobic mesophilic sludge digestion in wastewater treatment plants, but were ultimately killed in alkaline lime-treated stabilized sludge. The authors illustrated in lab scale tests that *Acinetobacter* spp. were not able to grow under anaerobic conditions but survived an incubation period of four weeks under the same conditions. The digestate of the anaerobic biogas process strain KPC-SM-21^T^ was isolated from represented the same type of environment. Retrospective studies have shown that *Acinetobacter* spp. accumulated efficiently intracellular polyphosphates, and thereby contributing to a minor extent to the phosphate elimination in sewage treatment plants (Fuhs and Chen 1975; Deinema et al. 1980, 1985; Wentzel et al. 1986; Bark et al. 1992; Van Groenestijn et al. 1987) reported that the accumulated polyphosphates in cells act as a phosphorus reserve and might be used as energy source by enzymatic processing of the polyphosphates via combined action of polyphosphate:AMP phosphotransferase and an adenylate kinase. Comparative genome analyses performed in EDGAR revealed the presence of genes that code for these enzymes in the KPC-SM-21^T^ genome (Fig. S11). This process could explain the survival of aerobic organisms in anaerobic biogas plant or anaerobic sludge treatment, because the polyphosphate reservoir in *Acinetobacter* cells can be vital under anaerobic environment conditions when these strict aerobes have no other source to generate energy (Kortstee et al. 1994).

## Conclusions

The reported phenotypic, chemotaxonomic, and genotypic characteristics congruently showed that KPC-SM-21^T^ (genomically highly similar to *Acinetobacter* sp. Marseille-Q1620 based on ANI value and core genome-based phylogeny) represents a novel species within the genus *Acinetobacter*, which is distinct from all hitherto described members of *Acinetobacter* at the species level of resolution. Next related species are *A. gerneri* (based on MLSA and core genome based phylogeny) and *A. baumannii* (based on 16S rRNA gene sequence identity). Although the physiological and molecular analyses revealed that *A. gerneri* CIP 107464^T^ = DSM 14967^T^ = KCTC 12415^T^ was next related to KPC-SM-21^T^, these two taxonomic entities were unequivocally different and distant from each other at the level of species based on all characteristics studied above. The name *Acinetobacter stercoris* sp. nov. is proposed, which indicates, that the bacterium was isolated from output manure of a biogas plant. The type strain is KPC-SM-21^T^ (= DSM 102168^T^ = LMG 29413^T^).

### **Description of*Acinetobacter stercoris*sp. nov.**

*Acinetobacter stercoris* (ster´co.ris. L.N. stercus faeces; L. gen. n. stercoris of manure, referring to the source of the isolate).

Cells are Gram-negative, oxidase negative, catalase positive, non-hemolytic, non-motile and coccobacilli. The optimum growth temperature is 25–37 °C; growth occurs at 45 °C and 10 °C, but not at 50 °C and 4 °C. Good growth occurred at 28 °C after 24 h on TS agar, R2A, NA, malt, Gly/Arg, CASO, K7, M65, DEV, LB, PYE, NU, and SBA. Very weak growth on MA, and no growth on MacConkey agar was observed. Tests for nitrate reduction, indole production, fermentation of d-glucose, urease activity, beta-galactosidase activity, esculin and gelatin hydrolysis were negative (result from API 20 NE). No acid production from d-sucrose, d-mannitol, dulcitol, d-salicin, adonitol, i-inositol, d-sorbitol, a-d-raffinose, α-l-rhamnose, d-maltose, d-trehalose, 1-O-Methyl-d-Glucosidpyranosid, i-erythritol, and d-arabitol. Acid was produced from α-d-glucose, α-d-lactose, l-arabinose, d-xylose, d-cellobiose, α-d-melibiose and d-mannose. Strong assimilation of N-acetyl-d-galactosamine, acetate, propionate, adipate, 4-aminobutyrate, fumarate, glutarate, Dl-lactate, l-malate, 2-oxoglutarate, pyruvate, l-alanine, l-aspartate, l-histidine, l-phenylalanine, l-proline, l-tryptophan, and 4-hydroxybenzoate, and weak assimilation of d-trehalose and (DL-3-) phenylacetate was observed, respectively. No assimilation of N-acetyl-d-glucosamine, p-arbutin, d-cellobiose, d-fructose, d-galactose, d-maltose, d-mannose, α-d-melibiose, (α-) l-rhamnose, d-sucrose, adonitol, I-inositol, maltitol, d-mannitol, d-sorbitol, DL-3-hydroxybutyrate, mesaconate, l-ornithine and 3-hydroxybenzoate, N-acetyl-glucosamine, and potassium gluconate, (d-) gluconate, (α-) d-glucose, d-ribose, d-salicin, putrescine, trans-aconitate, l-leucine and l-serine. Weak assimilation of l-arabinose, d-xylose, cis-aconitate, azelate, and suberate. Strong assimilation of citrate, itaconate, β-alanine, capric acid, adipic acid, d-malate (malic acid), citrate, and phenylacetic acid. No hydrolysis of p-nitrophenyl-β-d-galactopyranoside, p-nitrophenyl-β-d-glucuronide, p-nitrophenyl-α-d-glucopyranoside, p-nitrophenyl-phenyl-phosphonate, p-nitrophenyl-phosphate-disodium salt and l-proline-p-nitroanilide, p-nitrophenyl-β-d-xylopyranoside, bis-p-nitrophenyl-phosphate and l-glutamate-γ-carboxy-p-nitroanilide. However, hydrolysis of p-nitrophenyl-β-d-glucopyranoside and p-nitrophenyl-phosphoryl-choline was positive. Major fatty acids were C_18:1_ ω9c, C_16:0_ and summed feature 3* (containing C_16:1_ ω7c and/or iso-C_15:0_ 2-OH that was not determined by MIDI system).

The type strain KPC-SM-21^T^ (= DSM 102168^T^ = LMG 29413^T^) was isolated from the digestate of a biogas plant, located in the North of Hesse, Germany. The genomic DNA G + C content is 37.7 mol%. The NCBI/GenBank accession numbers for the whole draft genome sequence and partial 16S rRNA, *rpoB*, *gyrB* and housekeeping genes used in MLSA of KPC-SM-21^T^ were OOGT00000000, MT138756 and MT157622-MT157720, respectively. The complete sequences of 16S rRNA, *rpoB* and *gyrB* genes were also provided in the whole genome [16S rRNA (GenBank: OOGT01000238.1; Locus tag: KPC_R004), *rpoB* (GenBank: OOGT01000016, Locus tag: KPC_0582) and *gyrB* (GenBank: OOGT01000207.1, Locus tag: KPC_3210)].

## Supplementary Information

Below is the link to the electronic supplementary material.

Supplementary material 1 (PDF 1738 kb)

## Abbreviations

MALDI-TOF: Matrix assisted laser desorption/ionization time-of-flight
ANI: Average nucleotide identity
MLSA: Multilocus sequence analysis
