# Supplementary material for: Acinetobacter stercoris sp. nov. isolated from output source of a mesophilic german biogas plant with anaerobic operating conditions
Source: Antonie Van Leeuwenhoek. 2021 Feb 16;114(3):235–51. doi: 10.1007/s10482-021-01517-7 (PMC7902594; doi:10.1007/s10482-021-01517-7)
Supplement: Supplementary file 1 — Supplementary material 1 (PDF 1738 kb) [file 10482_2021_1517_MOESM1_ESM.pdf]

Supporting information

***Acinetobacter stercoris* sp. nov. isolated from output source of a mesophilic German biogas plant consisting anaerobic operating condition**

Dipen Pulami[1], Thorsten Schauss[1], Tobias Eisenberg[2], Jochen Blom[3], Oliver Schwengler[3], Jennifer K. Bender[4], Gottfried Wilharm[5], Peter Kämpfer[1], Stefanie P. Glaeser[1*]

Authors affiliation:

[1]Institut für Angewandte Mikrobiologie, Justus-Liebig-Universität Giessen, D-35392 Giessen, Germany

[2]Hessian State Laboratory, Department of Veterinary Medicine, Giessen, Germany

[3]Institute for Bioinformatics and Systems Biology, Giessen, D-35392 Giessen, Germany

[4]Division of Nosocomial Pathogens and Antibiotic Resistances, Robert Koch Institute, Wernigerode Branch, D-38855 Wernigerode, Germany

[5]Project group P2, Robert Koch Institute, Wernigerode Branch, D-38855 Wernigerode, Germany

***Corresponding author**:

Stefanie P. Glaeser

Stefanie.Glaeser@umwelt.uni-giessen.de

Supplementary Tables

Table S1 Genome accession and assembly number of organisms (including type species and strain) used in this study.

**Strain**	**GenBank Accession**	**GenBank assembly accession**
Acinetobacter sp. Marseille-Q1620	LR782267	GCA_902825285.1
Acinetobacter lwoffii NCTC 5866^T = CIP 64.10^T _= NIPH 512^T	APQS00000000	GCA_000369105.1
Acinetobacter baumannii ATCC 19606^T = CIP 70.34^T = JCM 6841^T	APRG00000000	GCA_000369385.1
Acinetobacter baylyi DSM 14961^T = CIP 107474^T	APPT00000000	GCA_000368685.1
Acinetobacter bereziniae LMG 1003^T = CIP 70.12^T	APQG00000000	GCA_000368925.1
Acinetobacter bohemicus ANC 3994^T	APOH00000000	GCA_000367925.1
Acinetobacter bouvetii DSM 14964^T = CIP 107468^T	APQD00000000	GCA_000368865.1
Acinetobacter brisouii CIP 110357	AYEU00000000	GCA_000488275.1
Acinetobacter calcoaceticus DSM 30006^T	APQI00000000	GCA_000368965.1
Acinetobacter equi 114^T	CP012808	GCA_001307195.1
Acinetobacter gandensis ANC 4275^T	LZDS00000000	GCA_001678755.1
Acinetobacter dijkshoorniae JVAP01^T	NZ_LJPG00000000	GCF_001595745.1
Acinetobacter gerneri DSM 14967^T = CIP 107464^T	APPN00000000	GCA_000368565.1
Acinetobacter guillouiae CIP 63.46^T	APOS00000000	GCA_000368145.1
Acinetobacter haemolyticus CIP 64.3^T	APQQ00000000	GCA_000369065.1
Acinetobacter harbinensis HITLi 7^T	JXBK00000000	GCA_000816495.1
Acinetobacter indicus CIP 110367^T = DSM 25388^T	BBSF00000000	GCA_000830155.1
Acinetobacter johnsonii CIP 64.6^T	APON00000000	GCA_000368045.1
Acinetobacter junii CIP 107470^T	APPS00000000	GCA_000368665.1
Acinetobacter junii CIP 64.5^T	APPX00000000	GCA_000368765.1
Acinetobacter lactucae NRRL B 41902^T	LRPE00000000	GCA_001605885.1
Acinetobacter nectaris CIP 110549	AYER00000000	GCA_000488215.1
Acinetobacter nosocomialis NIPH 2119^T	APOP00000000	GCA_000368085.1
Acinetobacter parvus DSM 16617^T	APOM00000000	GCA_000368025.1
Acinetobacter pittii ATCC 19004^T = CIP 70.29^T	APQP00000000	GCA_000369045.1
Acinetobacter radioresistens DSM 6976^T = CIP 103788^T	APQF00000000	GCA_000368905.1
Acinetobacter rudis DSM 24031^T	BBRX01000000	GCA_000829675.1
Acinetobacter schindleri CIP 107287	APPQ00000000	GCA_000368625.1
Acinetobacter seifertii NIPH 973^T	APOO00000000	GCA_000368065.1
Acinetobacter soli KCTC 22184^T	BBNM00000000	GCA_000760595.1
Acinetobacter tandoii DSM 14970^T = CIP 107469^T	AQFM00000000	GCA_000400735.1
Acinetobacter tjernbergiae DSM 14971^T = CIP 107465^T	AYEV00000000	GCA_000488175.1
Acinetobacter towneri DSM 14962^T = CIP 107472^T	APPY00000000	GCA_000368785.1
Acinetobacter ursingii DSM 16037^T = CIP 107286^T	APQA00000000	GCA_000368825.1
Acinetobacter venetianus RAG 1^T = CIP 110063^T	APPO00000000	GCA_000368585.1
Acinetobacter albensis ANC 4874^T	FMBK01000000	GCA_900095025.1
Acinetobacter defluvii WCHA30^T	MAUF00000000	GCA_001704615.2
Acinetobacter piscicola LW15^T	NIFO00000000	GCA_002233755.1
Acinetobacter populi PBJ7^T	NEXX00000000	GCA_002174125.1
Acinetobacter larvae BRTC-1^T	CP016895	GCA_001704115.1
Acinetobacter apis ANC 5114	FZLN00000000	GCA_900197575.1
"*Acinetobacter oleivorans* DR1 = KCTC 23045"	BCUK00000000	GCA_001591245.1
Moraxella lacunata NBRC 102154^T = CCUG 444^T	BCUK00000000	GCA_001591245.1

Table S2 Potential virulence factor related genes of strain KPC-SM-21^T. The genome of *A. baumannii* ATCC19606^T was used as reference. Analyses were performed in EDGAR 2.3 (Blom et al. 2016) and VFDB (virulence factor database; http://www.mgc.ac.cn/VFs/) which was used to indentify virulence related genes.

					Strain	
Gene/Locus/Operon	Protein	Predicted function	Role in pathogenesis	Reference for evidence	ATCC 19606T	KPC-SM-21^T
**Type 2 Secretion system**					Locus tag	
gspN	GspN	Unknown function	In vivo survival	Johnson et al. 2016; Elhosseiny et al. 2016; Harding et al. 2016; Harding, Hennon and Feldman 2018	HMPREF0010_01960	KPC_2986
gspC	GspC	Inner-membrane platform protein			HMPREF0010_01959	KPC_2987
gspD	GspD	Outer-membrane secretion			HMPREF0010_01958	KPC_2988
gspL	GspL	Inner-membrane platform protein			HMPREF0010_00073	KPC_0719
gspM	GspM	Inner-membrane platform protein			HMPREF0010_00074	KPC_0720
gspE	GspE	Secretion ATPase			HMPREF0010_01637	KPC_1813
gspF	GspF	Inner-membrane platform protein			HMPREF0010_01870	KPC_2178
gspKJIH	GspKJIH	Minor pseudopilins			HMPREF0010_00793-00796	KPC_2625-2628
**Type 6 secretion system**					Locus tag	
tssL	TssL	membrane complex proteins	interspecies competition, host colonization	Weber et al. 2013; Carruthers et al. 2013; Wright et al. 2014; Jones et al. 2015; Repizo et al. 2015	HMPREF0010_01111	KPC_3150
tssK	TssK	baseplate components			HMPREF0010_01112	KPC_3149
tssA	TssA	Priming protein			HMPREF0010_01113	KPC_3148
tssH	TssH	ATPase ClpV			HMPREF0010_01114	KPC_3147
tagN	TagN	Structural component			HMPREF0010_01116	KPC_0890
tagF	TagF	Posttranslational repressor of T6SS			HMPREF0010_01117	KPC_0889
tssM	TssM	membrane complex proteins			HMPREF0010_01118	KPC_0888
tssG	TssG	baseplate components			HMPREF0010_01120	KPC_0886
tssF	TssF	baseplate components			HMPREF0010_01121	KPC_0885
tssE	TssE	baseplate components			HMPREF0010_01122	KPC_0884
hcp	Hcp	tubule protein			HMPREF0010_01123	KPC_0883
tssB	TssB	Sheath components			HMPREF0010_01125	KPC_0881
tssC	TssC	Sheath components			HMPREF0010_01124	KPC_0882
**Others**					Locus tag	
OmpA (Omp38)	Outer-membrane protein A	Outer membrance protein	Adherence, invasion, apoptosis, biofilm, persistence	Choi et al. 2005; 2008a, 2008b; Gaddy, Tomaras and Actis 2009; Lee et al. 2010; Smani et al. 2014; Wang et al. 2014	HMPREF0010_02782	KPC_1415
ptK	protein tyrosine kinase	Capsular polymerisation and assembly	Biofilm, tissue infection, serum growth	Russo et al. 2010	HMPREF0010_03290	KPC_2580
epsA	putative polysaccharide export outermembrane protein	Capsular polymerisation and assembly	Human serum resistance, in vivo survival	Russo et al. 2010	HMPREF0010_03288	KPC_2582
nfuA	Fe/S protein NfuA	iron-sulfur cluster biogenesis in iron deficiency	survival in vivo, hosts cell attack, persistence	Zimbler et al. 2012	HMPREF0010_01516	KPC_3562
plC1	Phospholipase C	catalysing the cleavage of phospholipids present in host cell membrance aiding cell lysis enzymatic catalysis		Camarena et al 2010	HMPREF0010_03297	KPC_3412
plD	Phospholipase D/cardiolipin synthase			Jacobs et al 2010	HMPREF0010_00607	KPC_2840
cpaA	metalloprotease CpaA	Inhibition of blood coagulation		Tilley et al. 2014; Kinsella et al. 2017	absent	KPC_0460

Table S3 Potential phage genes of strain KPC-SM-21^{T}. Contig sequences were examined for phage related genes using PHASTER (https://phaster.ca/; Zhou et al. 2011; Arndt et al. 2016).

No.	Contig Accession	Completeness	Score	Position	Most common phage	Phage accession
**1.**	NZ_OOGT01000171.1	Incomplete	10	124-5588	PHAGE_Acinet_YMC11/11/R3177	NC_041866
**2.**	NZ_OOGT01000144.1	Incomplete	20	58-9763	PHAGE_Acinet_AbP2	NC_041998
**3.**	NZ_OOGT01000254.1	Incomplete	10	797-4507	PHAGE_Burkho_Bcep176	NC_007497
**4.**	NZ_OOGT01000013.1	Incomplete	10	2434-8498	PHAGE_Acinet_Bphi_B1251	NC_019541
**5.**	NZ_OOGT01000018.1	Incomplete	30	5003-11252	PHAGE_Acidov_ACP17	NC_041997
**6.**	NZ_OOGT01000008.1	Intact	120	4305-38981	PHAGE_Acinet_YMC11/11/R3177	NC_041866

Supplementry Figures

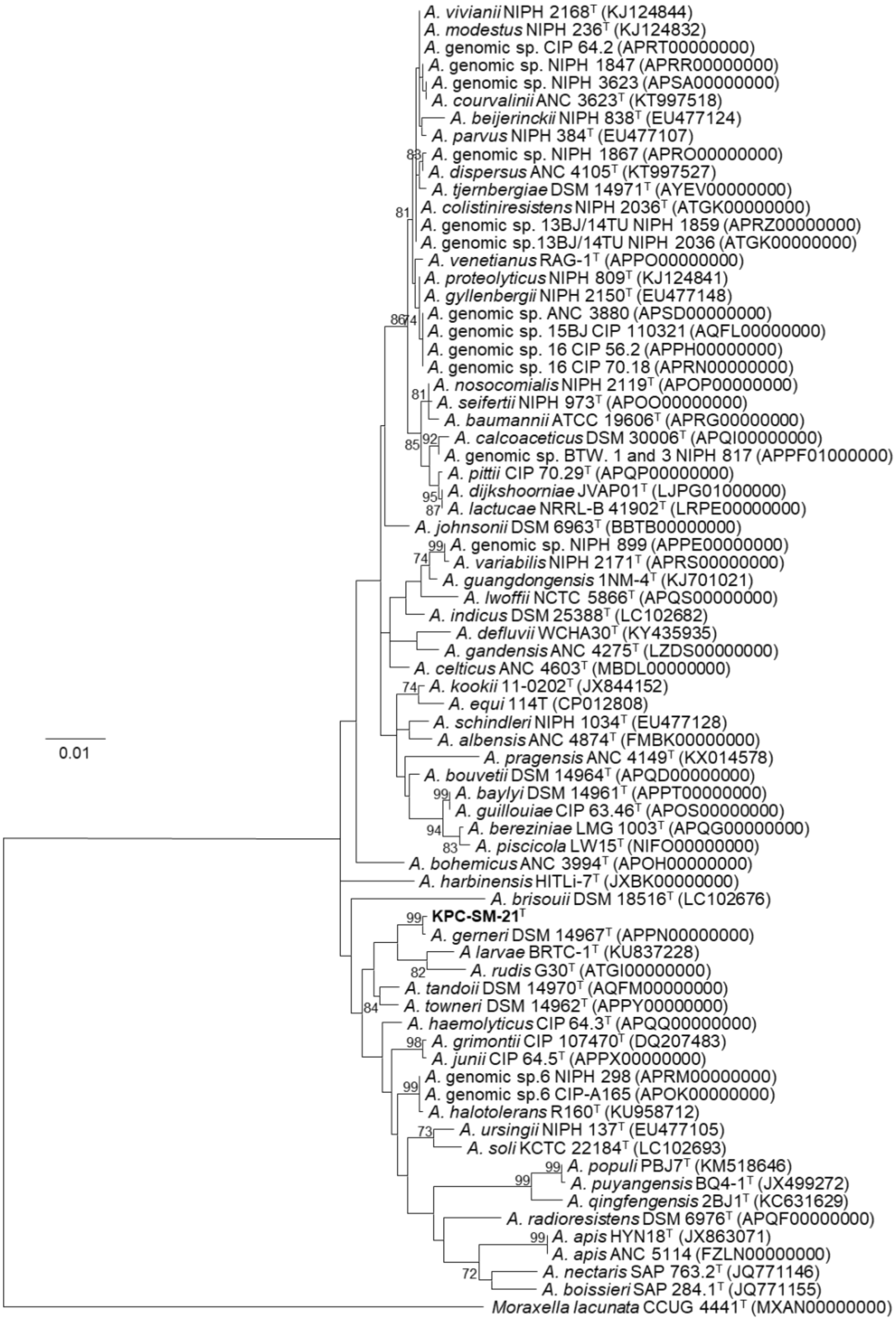

Fig. S1 Maximum-likelihood (Felsenstein 1981) tree based on aminoacid sequences of concatenated variable zones of *rpoB* gene, showing the placement of KPC-SM-21^T within the genus *Acinetobacter*. Bootstrap values (>70%) based on 100 replicates are shown at the branch nodes. *Moraxella lacunata* NBRC 102154^T was used as outgroup. Bar, 0.01 substitutions per site.

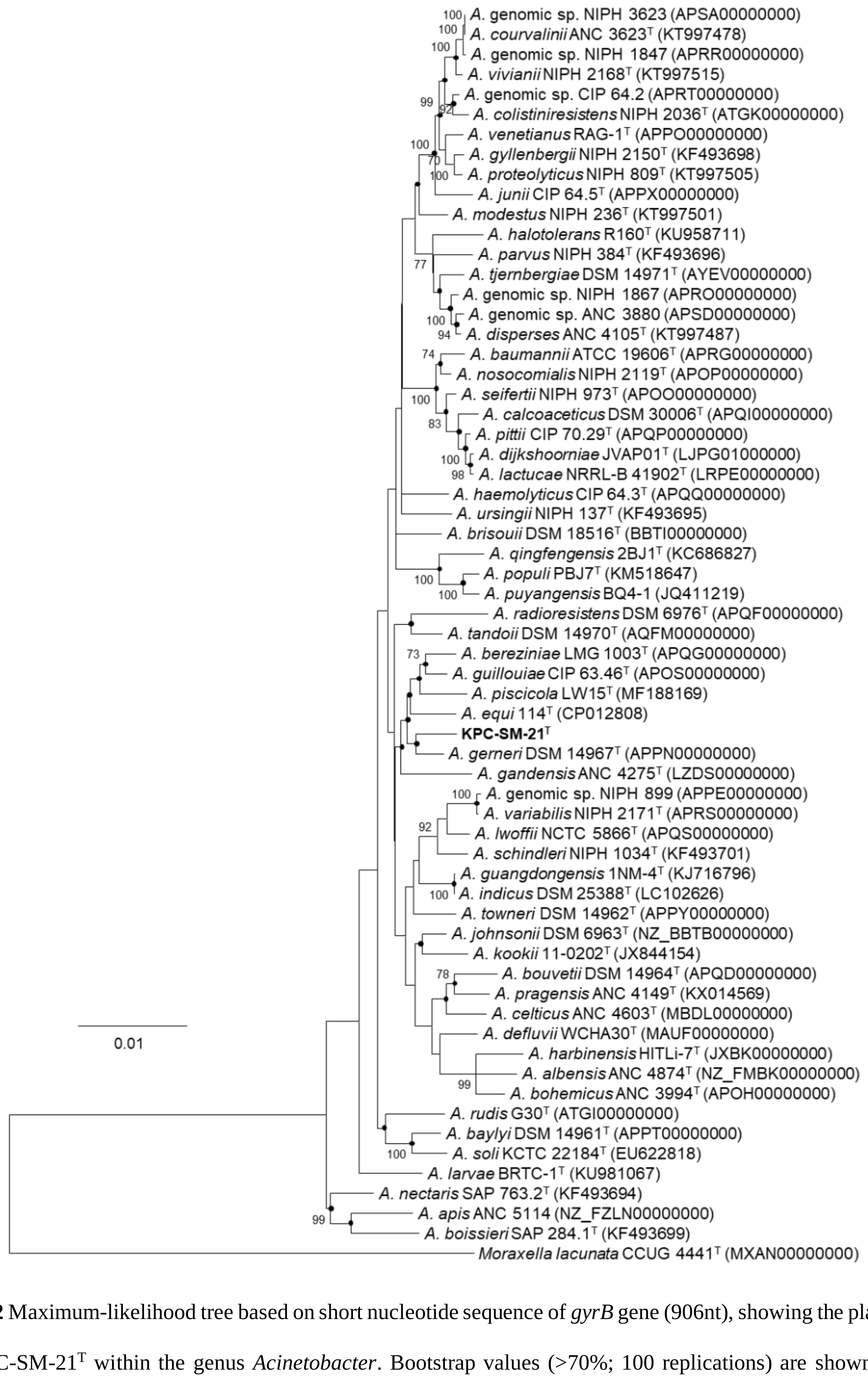

Fig. S2 Maximum-likelihood tree based on short nucleotide sequence of *gyrB* gene (906nt), showing the placement of KPC-SM-21^{T} within the genus *Acinetobacter*. Bootstrap values (>70%; 100 replications) are shown. Filled circles indicate nodes that were also present in tree generated by neighbour-joining method. *Moraxella lacunata* NBRC 102154^{T} was used as outgroup. Bar, 0.01 substitutions per site.

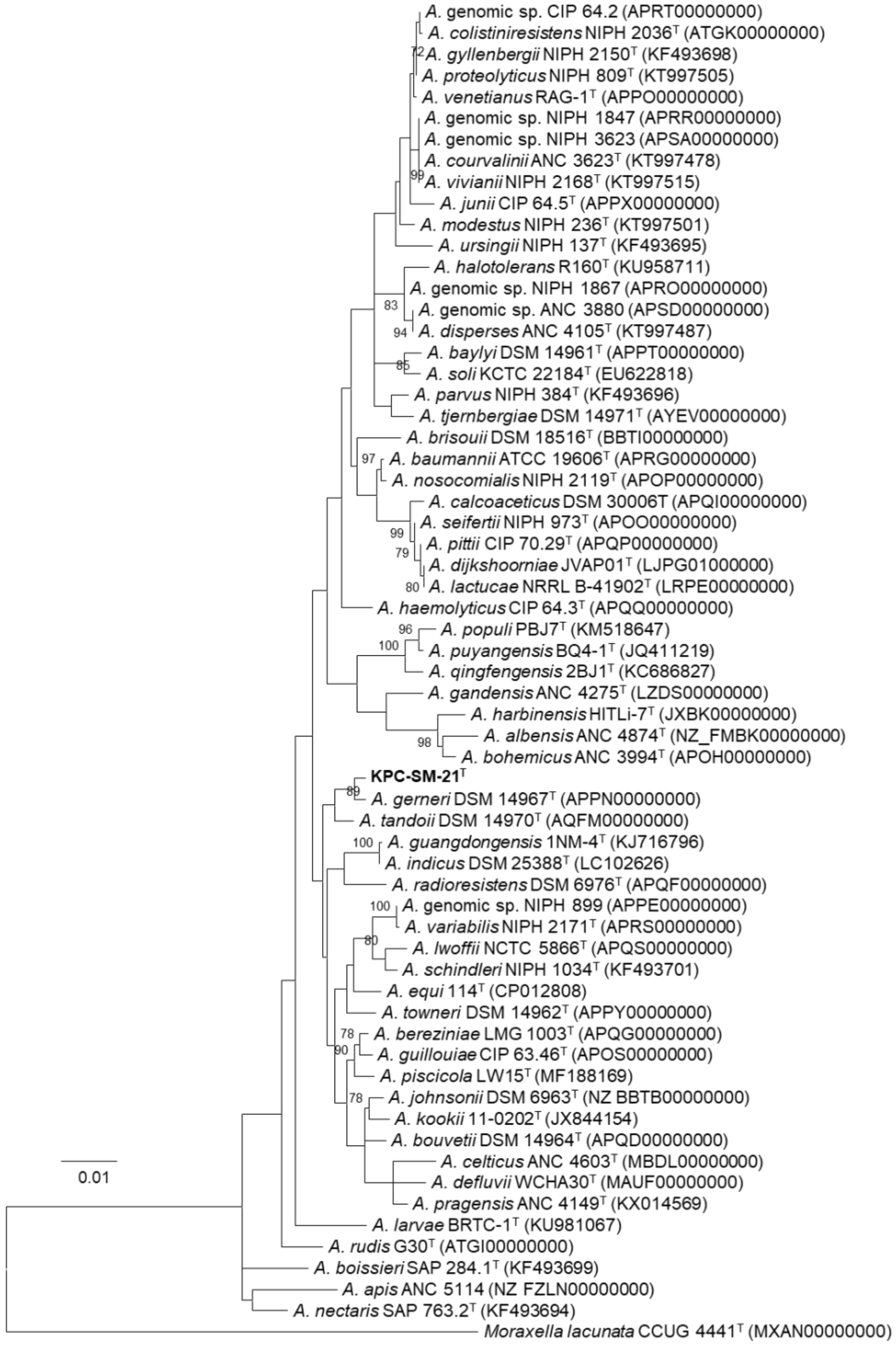

Fig. S3 Maximum-likelihood tree based on aminoacid sequence of *gyrB* gene, showing the placement of KPC-SM-21^{T} within the genus *Acinetobacter*. Bootstrap values (>70%) based on 100 replicates are shown at the branch nodes. *Moraxella lacunata* NBRC 102154^{T} was used as outgroup. Bar, 0.01 substitutions per site.

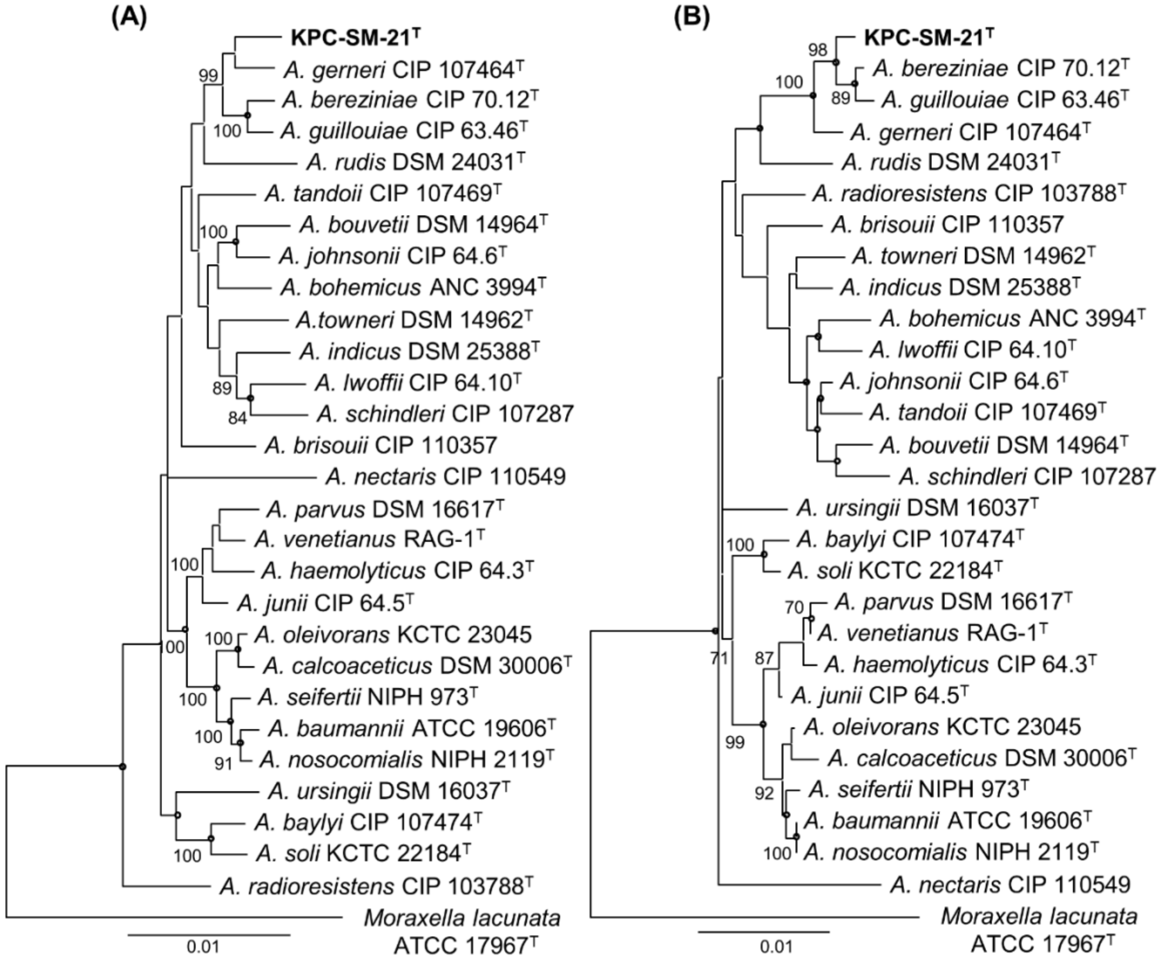

Fig. S4 Phylogenetic placement of strain KPC-SM-21^{T} within the genus *Acinetobacter* based on a six gene MLSA. The phylogenetic tree was calculated with the ML method and is based on concatenated partial *pyrG-cpn60-gltA-recA-rplB-rpoB* nucleotide (A) and respective amino acid (B). Bootstrap values of ≥70% (100 replications) are shown. Phylogenetic analyses were based on a total of 2,331 nucleotide and 777 amino acid codon positions. Filled circles indicate nodes that were also present in trees generated by NJ and maximum parsimony methods. *Moraxella lacunata* ATCC 17967^{T} was used as outgroup. Bars, 0.01% sequence divergence. Locus tag number of *pyrG, cpn60, gltA, recA, rplB and rpoB* genes of strain KPC-SM-21^{T} are KPC_1268, KPC_2174, KPC_3048, KPC_3432, KPC_0262, and KPC_0582.

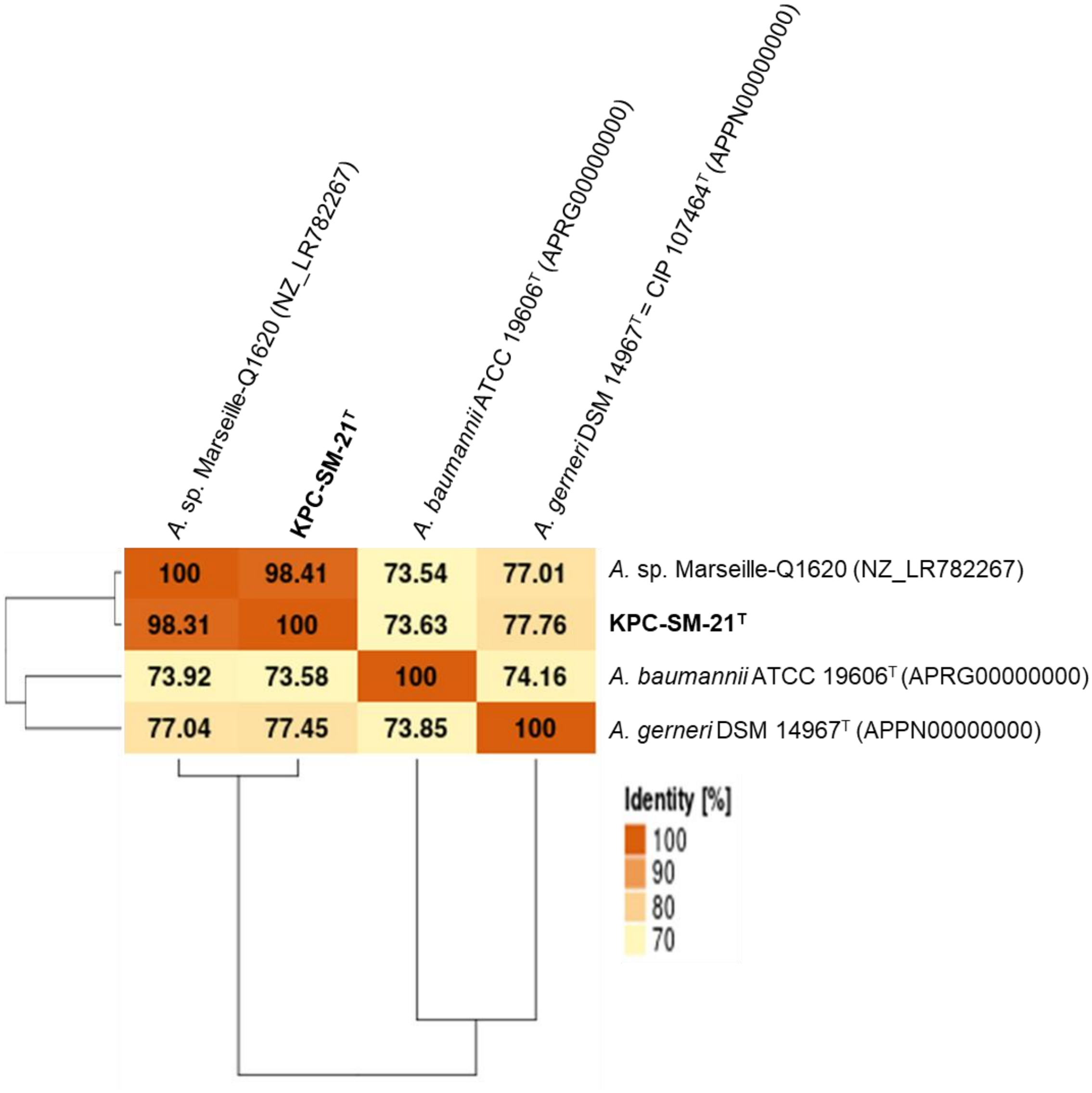

Fig. S5 Clustered heat map of ANI values between the strain KPC-SM-21^T, *Acinetobacter* sp. Marseille-Q1620, *A. gerneri* DSM 14967^T and *A. baumannii* ATCC 19606^T. The analyses were done in EDGAR 2.3 (Blom et al. 2016).

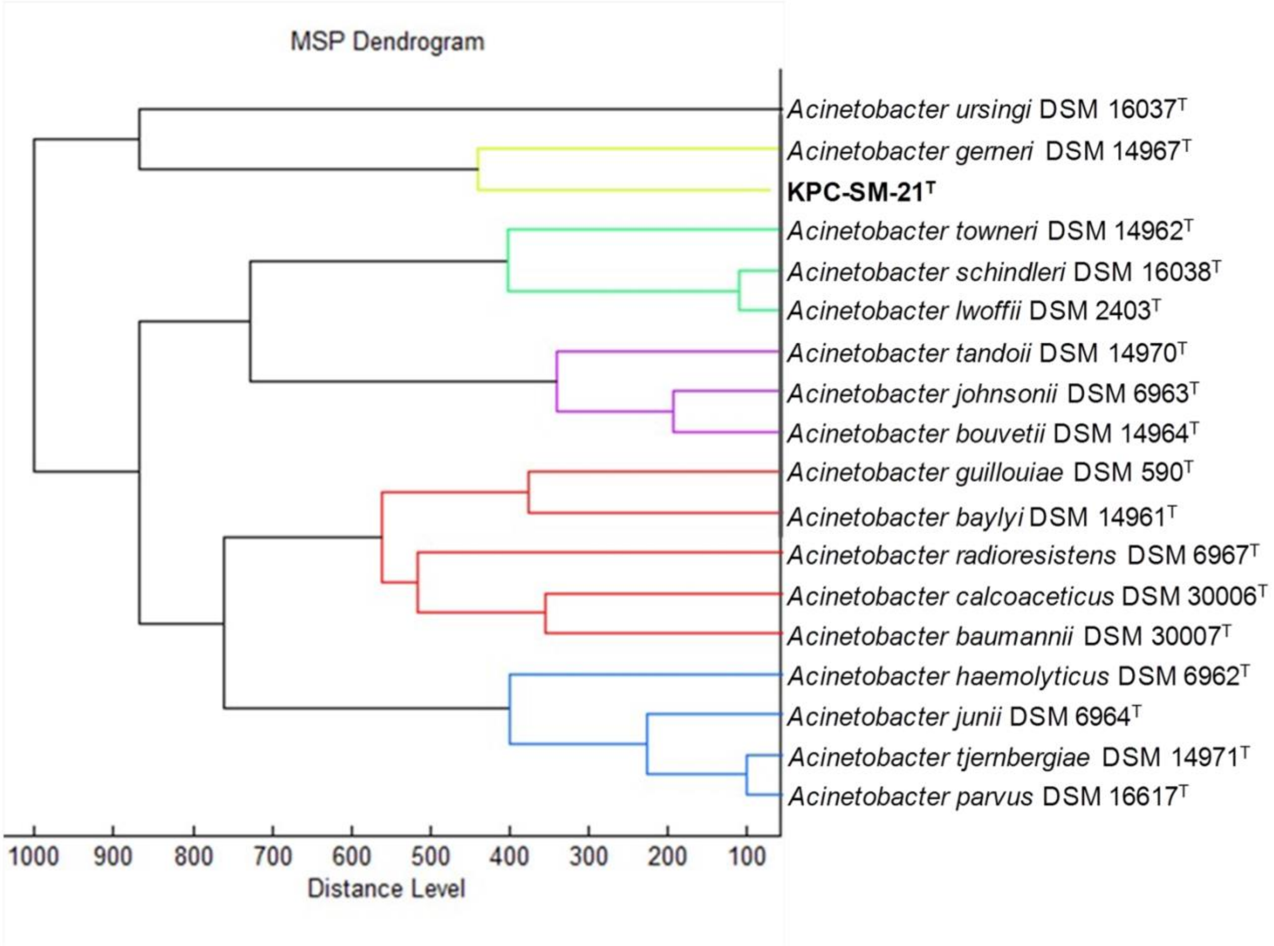

Fig. S6 Biotyper (Bruker Daltonics) generated dendrogram based on whole cell mass spectrometry (MALDI-TOF-MS) of KPC-SM-21^{T} (shown in bold font) and other type species of the genus *Acinetobacter*.

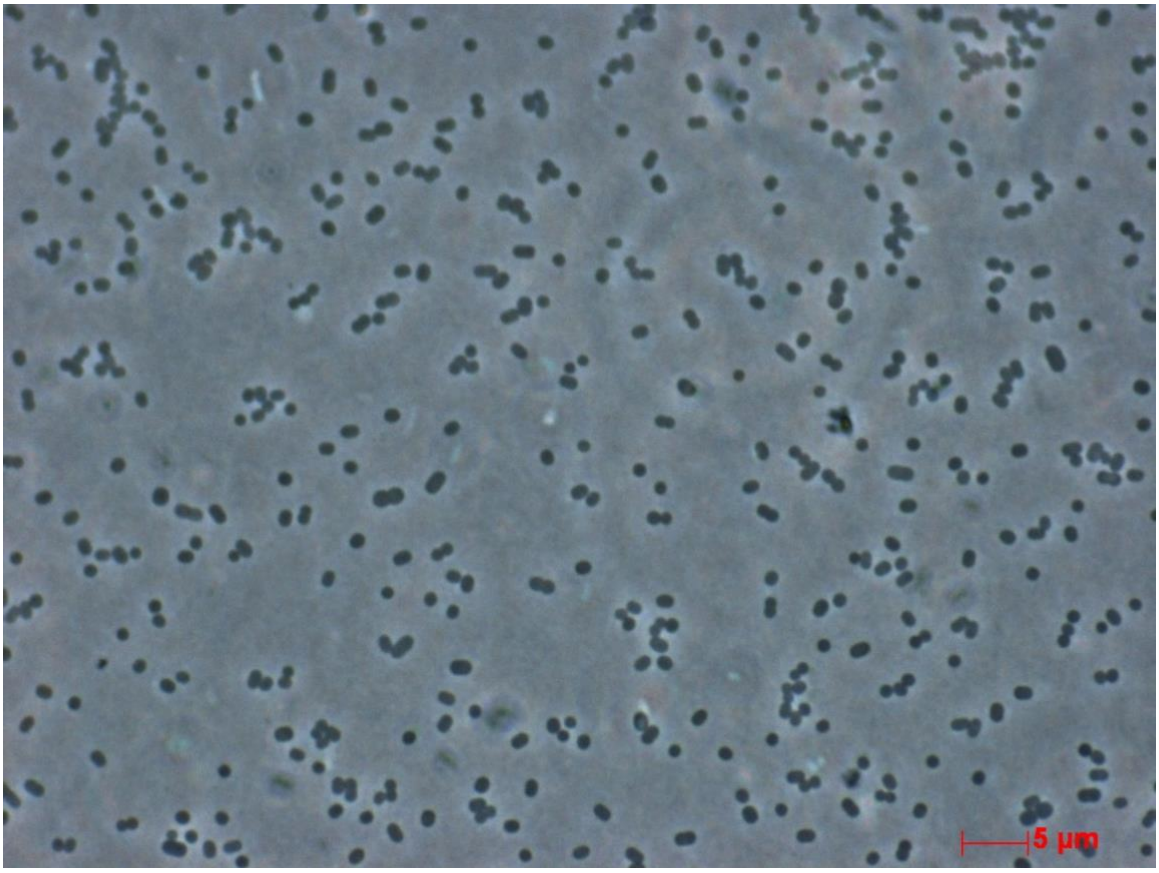

Fig. S7 Microsopical picture of cellls of strain KPC-SM-21^{T}. Size: length (1.45µm) × width (1.2µm), Gram - negative, typically short, rod shaped (coccobacilli: intermediated between spherical and rod shape), non motile.

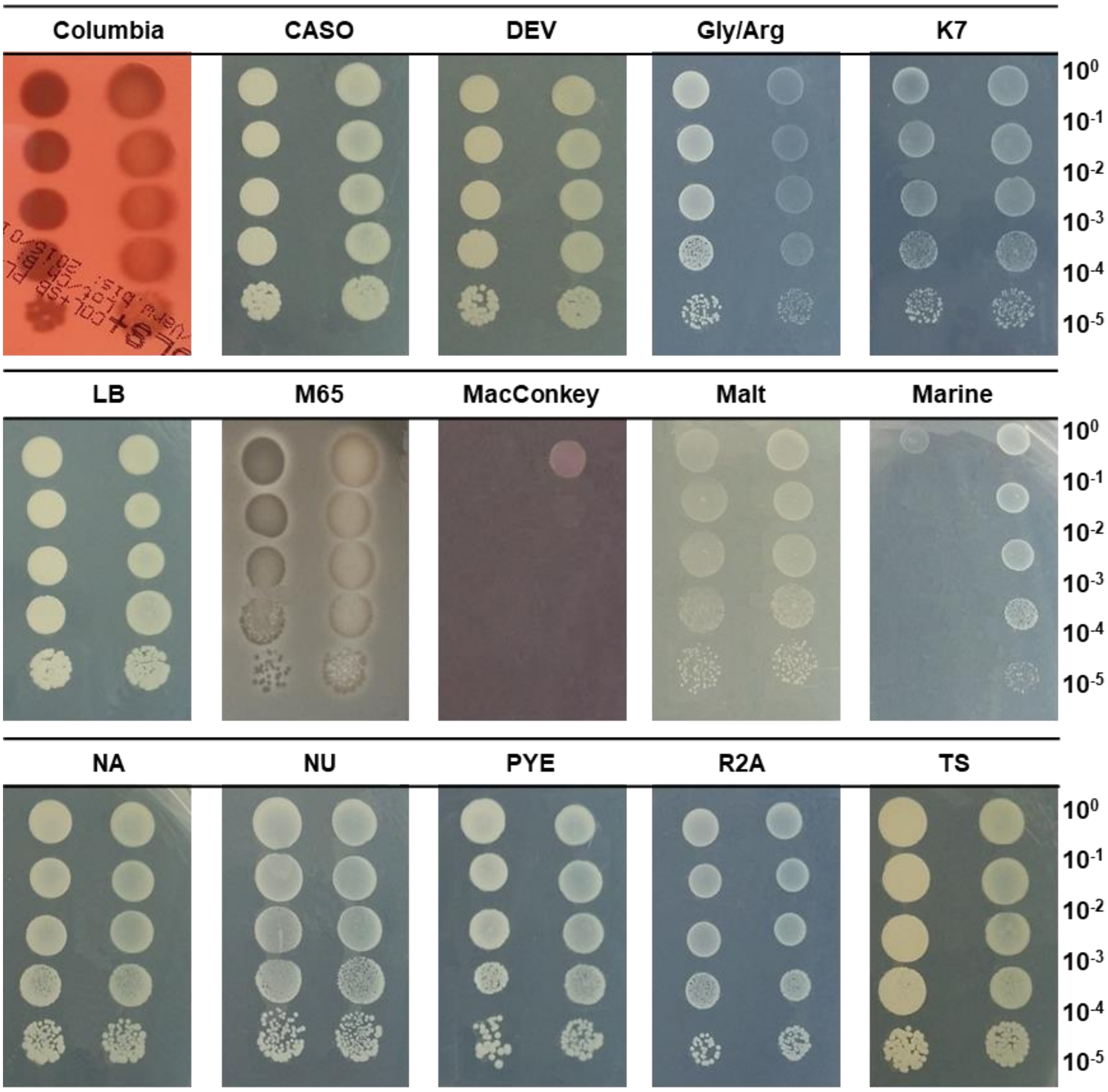

Fig. S8 Spot assay test showing growth of strain KPC-SM-21^T (left) and *A. baumannii* ATCC 19606^T (right) on different growth media. Serial dilutions (10^0 to 10^{-5}) of cell suspensions (McFarland 0.5, suspended in autoclaved 0.9% NaCl solution) were spotted on each plate (5 µL per spot) and incubated at 28°C for 7 days. Growth of KPC-SM-21^T occurred in all media except marine agar (weak growth) and MacConkey (no growth). Images were taken after 7 days of incubation.

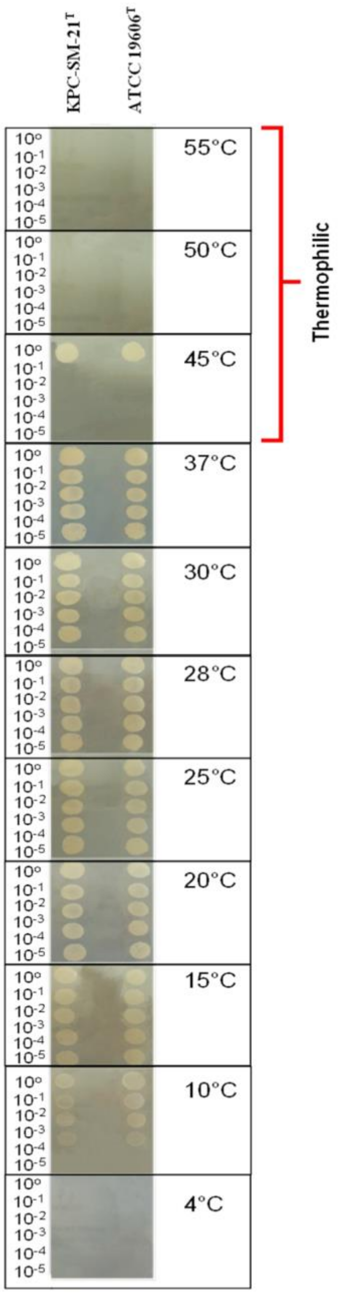

Fig. S9 Spot assay of temperature dependent growth of strain KPC-SM-21^{T} and *A. baumannii* ATCC 19606^{T} following 24h of incubation.

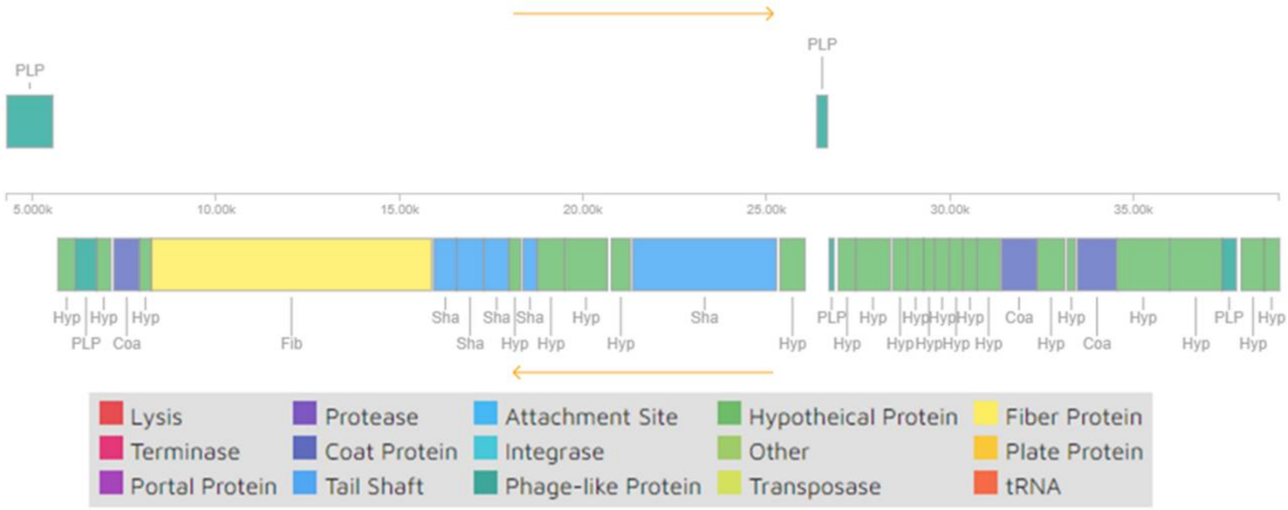

Fig. S10 Remnants of bacteriophage regions (Phage region 1) identified from querying the whole genome sequence of *Acinetobacter* sp. KPC-SM-21^{T} (Contig number: NZ_OOGT01000008.1) using PHASTER. The boxes are color coded with the legend provided below the figure to show their potential functions.

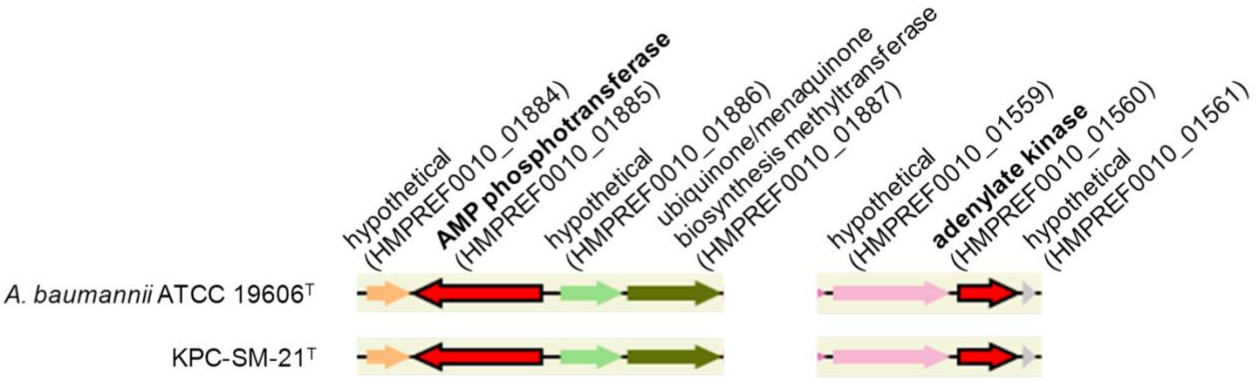

Fig. S11 Distribution of genes encoding AMP phosphotransferase (PPK2) and adenylate kinase (adk) in the genome of *A. baumannii* ATCC 19606^T (ACQB00000000) and KPC-SM-21^T, respectively. The locus tag of AMP phosphotransferase and adenylate kinase are KPC_0195 and KPC_2791 for KPC-SM-21^T. Horizontal arrows indicate the direction and orientation of the gene.

Reference

Arndt D, Grant JR, Marcu A, et al (2016) PHASTER: a better, faster version of the PHAST phage search tool. Nucleic Acids Res 44:W16–W21. https://doi.org/10.1093/nar/gkw387

Blom J, Kreis J, Spänig S, et al (2016) EDGAR 2.0: an enhanced software platform for comparative gene content analyses. Nucleic Acids Res 44:W22–W28. https://doi.org/10.1093/nar/gkw255

Camarena L, Bruno V, Euskirchen G, et al (2010) Molecular mechanisms of ethanol-induced pathogenesis revealed by RNA-sequencing. PLoS Pathog 6:1–14. https://doi.org/10.1371/journal.ppat.1000834

Carruthers MD, Nicholson PA, Tracy EN, Munson RS (2013) *Acinetobacter baumannii* utilizes a type VI secretion system for bacterial competition. PLoS One 8:. https://doi.org/10.1371/journal.pone.0059388

Choi CH, Hyun SH, Lee JY, et al (2008a) *Acinetobacter baumannii* outer membrane protein A targets the nucleus and induces cytotoxicity. Cell Microbiol 10:309–319. https://doi.org/10.1111/j.1462-5822.2007.01041.x

Choi CH, Lee EY, Lee YC, et al (2005) Outer membrane protein 38 of *Acinetobacter baumannii* localizes to the mitochondria and induces apoptosis of epithelial cells. Cell Microbiol 7:1127–1138. https://doi.org/10.1111/j.1462-5822.2005.00538.x

Choi CH, Lee JS, Lee YC, et al (2008b) *Acinetobacter baumannii* invades epithelial cells and outer membrane protein A mediates interactions with epithelial cells. BMC Microbiol 8:. https://doi.org/10.1186/1471-2180-8-216

Cui Y, Chen X, Luo H, et al (2016) BioCircos.js: An interactive Circos JavaScript library for biological data visualization on web applications. Bioinformatics 32:1740–1742. https://doi.org/10.1093/bioinformatics/btw041

Elhosseiny NM, El-Tayeb OM, Yassin AS, et al (2016) The secretome of *Acinetobacter baumannii* ATCC 17978 type II secretion system reveals a novel plasmid encoded phospholipase that could be implicated in lung colonization. Int J Med Microbiol 306:633–641. https://doi.org/10.1016/j.ijmm.2016.09.006

Felsenstein J (1981) Evolutionary trees from DNA sequences: A maximum likelihood approach. J Mol Evol 17:368–376. https://doi.org/10.1007/BF01734359

Gaddy JA, Tomaras AP, Actis LA (2009) The *Acinetobacter baumannii* 19606 OmpA protein plays a role in biofilm formation on abiotic surfaces and in the interaction of this pathogen with eukaryotic cells. Infect Immun 77:3150–3160. https://doi.org/10.1128/IAI.00096-09

Harding CM, Hennon SW, Feldman MF (2018) Uncovering the mechanisms of *Acinetobacter baumannii* virulence. Nat. Rev. Microbiol. 16:91–102

Harding CM, Kinsella RL, Palmer LD, et al (2016) Medically relevant *Acinetobacter* species require a type II secretion system and specific membrane-associated chaperones for the export of multiple substrates and full virulence. PLoS Pathog 12: https://doi.org/10.1371/journal.ppat.1005391

Jacobs AC, Hood I, Boyd KL, et al (2010) Inactivation of phospholipase D diminishes *Acinetobacter baumannii* pathogenesis. Infect Immun 78:1952–1962. https://doi.org/10.1128/IAI.00889-09

Johnson TL, Waack U, Smith S, et al (2016) *Acinetobacter baumannii* is dependent on the type II secretion system and its substrate LipA for lipid utilization and in vivo fitness. J Bacteriol 198:711–719. https://doi.org/10.1128/JB.00622-15

Jones CL, Clancy M, Honnold C, et al (2015) Fatal outbreak of an emerging clone of extensively drug-resistant *Acinetobacter baumannii* with enhanced virulence. Clin Infect Dis 61:145–154. https://doi.org/10.1093/cid/civ225

Kinsella RL, Lopez J, Palmer LD, et al (2017) Defining the interaction of the protease CpaA with its type II secretion chaperone CpaB and its contribution to virulence in *Acinetobacter* species. J Biol Chem 292:19628–19638. https://doi.org/10.1074/jbc.M117.808394

Lee JS, Choi CH, Kim JW, Lee JC (2010) *Acinetobacter baumannii* outer membrane protein a induces dendritic cell death through mitochondrial targeting. J Microbiol 48:387–392. https://doi.org/10.1007/s12275-010-0155-1

Repizo GD, Gagné S, Foucault-Grunenwald ML, et al (2015) Differential role of the T6SS in *Acinetobacter baumannii* virulence. PLoS One 10:. https://doi.org/10.1371/journal.pone.0138265

Russo TA, Luke NR, Beanan JM, et al (2010) The K1 capsular polysaccharide of *Acinetobacter baumannii* strain 307-0294 is a major virulence factor. Infect Immun 78:3993–4000. https://doi.org/10.1128/IAI.00366-10

Smani Y, Fàbrega A, Roca I, et al (2014) Role of OmpA in the multidrug resistance phenotype of *Acinetobacter baumannii*. Antimicrob Agents Chemother 58:1806–1808. https://doi.org/10.1128/AAC.02101-13

Tilley D, Law R, Warren S, et al (2014) CpaA a novel protease from *Acinetobacter baumannii* clinical isolates deregulates blood coagulation. FEMS Microbiol Lett 356:53–61. https://doi.org/10.1111/1574-6968.12496

Wang N, Ozer EA, Mandel MJ, Hauser AR (2014) Genome-wide identification of *Acinetobacter baumannii* genes necessary for persistence in the lung. MBio 5:. https://doi.org/10.1128/mBio.01163-14

Weber BS, Miyata ST, Iwashkiw JA, et al (2013) Genomic and functional analysis of the type VI secretion system in *Acinetobacter*. PLoS One 8:. https://doi.org/10.1371/journal.pone.0055142

Wright MS, Haft DH, Harkins DM, et al (2014) New insights into dissemination and variation of the health care-associated pathogen *Acinetobacter baumannii* from genomic analysis. MBio 5:. https://doi.org/10.1128/mBio.00963-13

Zhou Y, Liang Y, Lynch KH, et al (2011) PHAST: A Fast Phage Search Tool. Nucleic Acids Res 39:. https://doi.org/10.1093/nar/gkr485

Zimbler DL, Park TM, Arivett BA, et al (2012) Stress response and virulence functions of the *Acinetobacter baumannii* NfuA Fe-S scaffold protein. J Bacteriol 194:2884–2893. https://doi.org/10.1128/JB.00213-12
